# Characterisation of a new online nanoLC-CZE-MS platform and application for the glycosylation profiling of alpha-1-acid glycoprotein

**DOI:** 10.1007/s00216-021-03814-6

**Published:** 2021-12-09

**Authors:** Alexander Stolz, Christian Neusüß

**Affiliations:** 1 Faculty of Chemistry, Aalen University, Beethovenstr. 1, 73430 Aalen, Germany; 2 Department of Pharmaceutical and Medicinal Chemistry, Friedrich Schiller University, 07743 Jena, Germany

**Keywords:** Capillary zone electrophoresis, Glycoprotein, Heart-cut, Intact protein analysis, Orosomucoid, Two-dimensional separation

## Abstract

The ever-increasing complexity of biological samples to be analysed by mass spectrometry has led to the necessity of sophisticated separation techniques, including multidimensional separation. Despite a high degree of orthogonality, the coupling of liquid chromatography (LC) and capillary zone electrophoresis (CZE) has not gained notable attention in research. Here, we present a heart-cut nanoLC-CZE-ESI-MS platform to analyse intact proteins. NanoLC and CZE-MS are coupled using a four-port valve with an internal nanoliter loop. NanoLC and CZE-MS conditions were optimised independently to find ideal conditions for the combined setup. The valve setup enables an ideal transfer efficiency between the dimensions while maintaining good separation conditions in both dimensions. Due to the higher loadability, the nanoLC-CZE-MS setup exhibits a 280-fold increased concentration sensitivity compared to CZE-MS. The platform was used to characterise intact human alpha-1-acid glycoprotein (AGP), an extremely heterogeneous N-glycosylated protein. With the nanoLC-CZE-MS approach, 368 glycoforms can be assigned at a concentration of 50 μg/mL as opposed to the assignment of only 186 glycoforms from 1 mg/mL by CZE-MS. Additionally, we demonstrate that glycosylation profiling is accessible for dried blood spot analysis (25 μg/mL AGP spiked), indicating the general applicability of our setup to biological matrices. The combination of high sensitivity and orthogonal selectivity in both dimensions makes the here-presented nanoLC-CZE-MS approach capable of detailed characterisation of intact proteins and their proteoforms from complex biological samples and in physiologically relevant concentrations.

**Graphical abstract:**

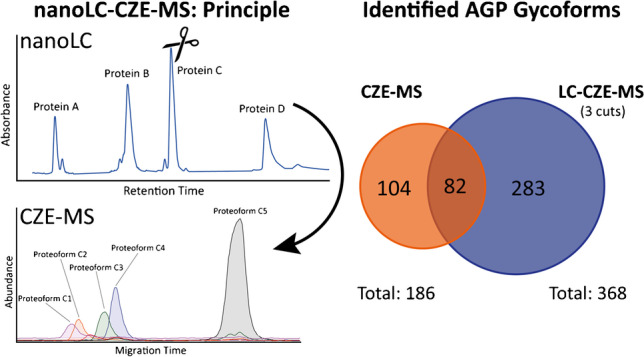

**Supplementary Information:**

The online version contains supplementary material available at 10.1007/s00216-021-03814-6.

## Introduction

Mass spectrometry (MS) has developed into an indispensable tool in various fields, including the analysis of biological and pharmaceutical proteins [1, 2]. MS offers high sensitivity and the possibility of compound identification, and in the field of proteomics, it allows sequencing and characterisation of peptides and intact proteins. To fully exploit the capabilities of MS, front-end separation is essential to reduce ion suppression, resolve compounds with identical or similar masses and boost sensitivity. Although major advances have been made to improve the separation of peptides and intact proteins by liquid chromatography (LC) and capillary zone electrophoresis (CZE), respectively, one-dimensional separation is often insufficient to adequately characterise complex samples [3, 4].

To increase separation capabilities, multidimensional separation platforms, primarily two-dimensional (2D), are increasingly applied in proteomics [5] and biopharmaceutical analysis [6]. 2D separations can either be performed offline or online. In offline techniques, a defined number of fractions are collected from the first dimension (^1^D) and are successively subjected to the second-dimension (^2^D) separation. This approach is technically easy to apply, sample treatment can be performed between the two separations and there are virtually no restrictions in the separation techniques to be coupled. However, automation is challenging, total analysis time tends to be long and significant sample loss occurs during the transfer from the ^1^D to the ^2^D. Online 2D separations, in which the ^1^D fraction is directly transferred to the ^2^D, can broadly be classified as either comprehensive or heart-cut techniques [7]. In comprehensive mode, the effluent from the ^1^D is completely sequentially transferred to the ^2^D. Moreover, the transfer frequency is high, allowing the transfer of multiple fractions per ^1^D peak, thereby retaining the separation of the ^1^D [8]. The comprehensive mode aims to maximise peak capacity to characterise complex mixtures. In the (multiple) heart-cut mode, only one or several fractions are transferred from the ^1^D to the ^2^D. Here, the main objective is to enable separation of selected analytes that are not resolved in the ^1^D. The limitation to the transfer of one or a few fractions allows independent selection and optimisation of separation conditions in both dimensions.

Due to the fundamentally different separation mechanisms, hence high orthogonality, the coupling of LC and CZE has been proposed as promising for a long time [9]. Despite the theoretical benefits of such an approach, the number of publications of LC-CZE couplings is limited. To transfer effluent from the ^1^D to the ^2^D in LC-CZE, different technical approaches were described. These include flow-gating [10–12], microfluidic [13, 14] and valve-based approaches [15–17]. Our group has used commercial, as well as custom-built, nanoliter valves to perform 2D CE separations of intact proteins, especially monoclonal antibodies [18–20]. The low internal volumes (4–40 nL), as well as the insulating material, makes these valves promising for LC-CZE coupling. Based on a proof-of-concept study, we demonstrated the general applicability of such a valve to couple nanoLC and CZE-MS [21]. A comprehensive review and comparison of LC-CZE systems, as well as relevant applications, was recently published by Ranjbar et al. [4]. Among the reported setups, most use optical detection. LC-CZE platforms using MS detection were only scarcely reported in literature [11–14, 22–24], and besides our own work, none of them describe the separation of intact proteins.

Considering the advent of top-down mass spectrometry (TDMS) in proteomics and biopharmaceutical analysis, sophisticated separation platforms are needed for intact proteins [25, 26]. Here, the combination of LC and CZE is particularly interesting as both separation techniques address different molecular characteristics of proteins. While the hydrophobicity-based mechanism of reversed-phase (RP) LC enables separation of proteins (due to different amino acid sequences), the electrophoretic mobility-based separation of CZE uniquely enables separation of proteoforms as many post-translational modifications (PTM) alter the number of potential charge carriers in the protein. These different characteristics can be used to characterise complex protein mixtures on the proteoform level (the term proteoform is used according to Smith and Kelleher) [27]. One example of such a complex proteoform mixture is the microheterogeneity of alpha-1-acid glycoprotein (AGP). AGP is an acute-phase plasma protein that is upregulated in response to inflammation and infection [28]. AGP is a 33–39-kDa protein and is highly glycosylated with a carbohydrate content of ~ 45% (w/w) and 5 potential N-glycosylation sites [29, 30]. The high content of sialic acids in the glycan structure results in a very low isoelectric point of 2.8–3.8 [28]. Additionally, the protein is present in several sequence variants [28]. This combination makes human AGP an extremely diverse protein with hundreds of proteoforms. The glycosylation profile is known to be influenced by various diseases [31–33], making detailed characterisation of this microheterogeneity clinically interesting.

Here, we present a novel nanoLC-CZE-MS platform for the analysis of intact proteins. The basic concept is based on our previous study. Here, we fully optimised and characterised a nanoLC-CZE-MS platform regarding the following aspects: The two dimensions were developed independently to obtain ideal separation capabilities. The platform was optimised towards a high transfer efficiency from ^1^D to ^2^D and was characterised regarding repeatability and sensitivity. This 2D platform was used to perform detailed profiling of glycosylation and sequence variants of human alpha-1-acid glycoprotein (AGP).

## Materials and methods

### Chemicals and material

Ultrapure water (UPW, 18 MΩ*cm at 25 °C) was prepared with an SG Ultra Clear UV (Siemens Water Technologies, USA). Acetonitrile (ACN, LC-MS grade), 2-propanol (LC-MS grade), acetic acid (HA) and formic acid (≥ 98%) (FA) were purchased from Carl Roth (Karlsruhe, Germany). Hydrochloric acid (37%), hydrofluoric acid (40%) and sodium hydroxide (1 M) were purchased from Merck KgaA (Darmstadt, Germany). Trifluoracetic acid (LiChrompur ≥ 99%), 4-(2-hydroxyethyl)-1-piperazineethanesulfonic acid (HEPES), alpha-1-acid glycoprotein from human plasma (≥ 99%), bovine serum albumin (BSA, ≥ 96%), cytochrome C (Cyt C) from equine heart (≥ 95%), lysozyme (Lys) from chicken egg white, myoglobin (Myo) from equine skeletal muscle (95–100%) and ribonuclease A (RNAse A) from bovine pancreas (≥ 60%) were all acquired from Sigma-Aldrich (Steinheim, Germany). Ribonuclease B from bovine pancreas was purchased from Abnova (Taipei, Taiwan). Poly(diallyldimethyl-ammonium) chloride (PDADMAC), 20% w/w in UPW (average MW: 500,000 g/mol) and poly(methacrylic acid) (PMA) were purchased from Sigma-Aldrich (Saint-Quentin Fallavier, France). Quarternized diethylaminoethyl dextran (DEAEDq) was obtained from Pharmacia (Uppsala, Sweden). FS capillaries with 30 and 50 μm inner diameter (ID) and 375 μm outer diameter were acquired from Polymicro Technologies (Phoenix, AZ, USA). Glass emitters with 750 μm ID and an orifice tip of 30 μm were obtained from BioMedical Instruments (Zoellnitz, Germany).

### Sample preparation

For method development and characterisation, a mixture containing RNAse A, Cyt C, Lys and Myo was used and prepared as follows: stock solutions of the proteins (4 mg/mL in UPW) were mixed to a final solution of 1 mg/mL per protein. The protein solution was divided into aliquots of 5–50 μL and dried by vacuum centrifugation for several hours at room temperature. The dried aliquots were stored at − 20 °C. Before usage, the aliquots were resolved with 0.1% (v/v) TFA solution to the final concentration for nanoLC and nanoLC-CZE-MS analysis. For CZE-MS, the aliquots were solved in 15% ACN + 0.1% TFA (if not stated otherwise, all percentages are v/v). Samples were used for a maximum of 5 days and stored at 4–8 °C. AGP aliquots were prepared similarly with a stock solution of 2 mg/mL.

Human dried blood spot (DBS) samples were obtained from Uppsala University Hospital (Sweden). All human samples used were leftovers after the completion of diagnostic tests and were used in accordance with the regional ethical review board of Uppsala (no 2001/367). DBS samples were prepared by punching out a 3-mm disk on a Whatmann mat. Proteins were extracted by incubation with 100 μL of 20 mM HEPES buffer (pH 7.4) for 30 min. After centrifugation, 1:10 or 1:20 dilutions (with 0.1% TFA) were prepared for analysis.

### CZE-MS

All capillaries were etched with hydrofluoric acid to reduce the outer diameter to < 150 μm as described elsewhere [34, 35]. PVA capillaries were prepared as described elsewhere [36]. SMIL-coated capillaries were prepared as previously described [37]. For this study, two different combinations of polycation and polyanion were used: PDADAMAC-PMA and DEAEDq-PMA. Capillaries were initially flushed (all flush steps with 2 bar pressure) for 20 min with 1 M sodium hydroxide, 5 min with UPW and 10 min with 20 mM HEPES buffer (pH 7.4). Subsequently, the capillaries were alternately flushed for 7 min with 3 mg/mL polycation (PDADMAC or DEAEDq) and polyanion (PMA) solutions with a 3-min 20-mM HEPES flush between each coating step. After attachment of the last polycation layer, the capillary was flushed with background electrolyte (BGE) for 10 min. To stabilise the coating before first use, − 10 kV was applied for 10 min.

For CZE-MS and nanoLC-CZE-MS, the following CZE-MS setup was used. CZE-MS was performed with a G1600 HP 3D CE instrument (Agilent Technologies, Waldbronn, Germany) on 50-μm-ID, 70-cm PVA, PDADMAC-PMA- or DEAEDq-PMA-coated capillaries. Before analysis, a capillary was flushed with BGE (0.2 M FA for the PDADMAC-PMA and 2 M HA for the DEAEDq-PMA coating) for 2 min followed by application of − 10 kV for 5 min. Hydrodynamic injection was performed for 10 s at 50 mbar. Separation was performed at + 30 kV for PVA, − 30 kV for the PDADMAC-PMA and − 15 kV for DEAEDq-PMA coating. For the nanoLC-CZE-MS coupling, − 15 kV was applied for both coatings. The CE instrument was coupled to a Bruker Compact QTOF MS (Bruker Daltonics, Bremen, Germany) via an in-house built nanoflow sheath liquid interface equipped with a 30-μm tip ID emitter [35]. 50:50 2-propanol with either 0.5% FA or 1% HA was used as sheath liquid (SL) for PDADMAC-PMAPVA-coated capillaries and DEAEDq-PMA-coated capillaries respectively. The spray voltage was set individually between 1700 and 2100 V for stable operation before each measurement. Dry temperature was set to 180 °C with a scan rate of 500–2500 *m*/*z* for the standard protein mix and 1500–3500 *m*/*z* for AGP. Lens voltages and other transfer parameters were optimised to the chosen *m*/*z* range respectively.

### Nano liquid chromatography

All separations were performed on an UltiMate™ 3000 RSLCnano system (Thermo Fisher, Germering, Germany). The system was coupled to an external ECD2600 EX UV detector (ECOM spol. S r.o., Prague, Czech Republic) by connecting the column end to a 30-μm-ID, 375-μm-OD FS capillary with a UV window (detection capillary). Detection was performed at 205 nm and 2 Hz scan rate. For all methods, eluent A was composed of 100% UPW + 0.1% TFA and eluent B of 80% ACN, 20% UPW + 0.1% TFA. For the separation of the protein mixture, three different columns were used: An Acclaim PepMap C18 column (75 μm × 150 mm) was obtained from Thermo Fisher Scientific and a C4 (75 μm × 500 mm) column from CoAnnTech Technologies (Richland, USA). PLRP-S (75 μm × 250 mm) columns were slurry-packed in-house. All columns were held at 60 °C during measurement. For each column, gradients were developed for optimised separation of proteins. All gradients for the three columns are described in the supporting information (SI)*.* After column comparison, the PLRP-S column (75 μm × 250 mm, 5 μm PLRP-S, 1000 Å) was chosen for all subsequent measurements. To allow the injection of higher sample volumes (up to 20 μL), a 150 μm × 50–60 mm trap column (C4, 3 μm, 300 Å) was used (CoAnnTech Technologies, Richland, USA). Separation was performed with a flow rate of either 300 nL/min or 100 nL/min with the following gradients:

For the 300-nL/min method, the trap column was loaded with 15% B at a flow rate of 4 μL/min for 10 min while the analytical column was equilibrated with 15% B at 500 nL/min flow rate. After loading, the column-switching valve was switched and separation was performed with a linear gradient of 32 min to 60% B at 300 nL/min. The column was flushed with 95% B for 7 min before re-equilibration at 15% B for 10 min. The overall method length was 60 min.

For the 100 nL/min method, loading was performed the same way while column equilibration was performed at 500 nL/min. After switching the valve, separation was performed with a linear gradient of 31 min to 60% B, followed by 17 min flushing at 95% B at 100 nL/min. Column equilibration was performed at 15% B for 10 min at 100 nL/min and another 5 min at 500 nL/min. The overall method length was 75 min.

### Data analysis

UV data were exported as *x*-*y* data from ECOMAC software (version 0.281, ECOM spol. S r.o., Prague, Czech Republic). A baseline correction was performed with a Matlab script as described in the SI. Calculation of peak area, height and full width at half maximum (FWHM) was performed with the baseline-corrected chromatogram in CE Val (version 0.6i2) [38]. MS data analysis was performed with DataAnalysis (version 4.3, Bruker Daltonics, Bremen, Germany). The *m*/z values to create the extracted ion electropherograms (EIE) for the different proteins and RNAse B glycoforms are summarised in Table S1 in the SI. Prior to integration, all EIEs were smoothed with a Gauss algorithm and 3 s smoothing width.

#### Calculation of peak volumes and transfer efficiency

The peak volumes (*V*_p_) of nanoLC peaks were calculated by the following formula:

1 $${V}_{\mathrm{p}}=2\ast 2.326\ast \frac{\mathrm{FWHM}}{2.355}\ast Q=1.975\ast \mathrm{FWHM}\ast Q$$

where ^˙^*Q* is the flow rate in nanoliters per minute and FWHM/2.355 is an expression for the standard deviation of the peak in the time domain in minutes. 2.326 is the *z*-value (two-sided) that corresponds to 98% of the peak integral assuming a Gaussian peak shape. Hence, the peak volume is estimated on a peak width corresponding to 98% peak integral assuming a Gaussian peak shape.

The transfer efficiency (TE_Vol_) regarding peak volume is calculated as follows:

2 $${\mathrm{TE}}_{\mathrm{vol}}=\frac{V_{\mathrm{loop}}}{V_{\mathrm{p}}}$$

Accordingly, the transfer efficiency regarding molar analyte amount is calculated by integration of the cut fraction of the peak assuming a Gaussian peak shape.

#### Analysis of AGP MS data

Transferred AGP fractions were analysed in PMI Intact Mass v3.4 (Protein Metrics, Cupertino, CA, USA). For each separation, time slices of 15 or 30 s were created to cover the peak containing the different AGP glycoforms. Mass spectra in these time slices were deconvoluted to obtain a mass list for each time slice. The obtained masses were matched to the theoretical masses with an allowed deviation of 0.5 Da. If one measured mass matched to multiple theoretical masses, the match with the lowest deviation was chosen. This results in a list of putative glycoform assignments.

## Results and discussion

### Optimisation of nanoLC and CZE conditions

To facilitate ideal conditions for the 2D setup, we optimised both dimensions independently regarding different aspects in 1D experiments. For the evaluation of LC parameters, a model protein mix of RNAse A, Cyt C, Lys and Myo in equivalent concentrations was used. For the optimisation of CZE conditions, RNAse B was used.

### LC conditions

Initial experiments were performed to evaluate ideal LC separation conditions to be used in the 2D setup. Three columns with different stationary phases and column dimensions were evaluated for intact protein analysis. Separation of the model proteins was possible on all three columns with varying separation efficiency. After initial gradient optimization for all columns, they were compared in terms of separation efficiency (*N*) and resolution (*R*_S_, Table 1). Both the CoAnn as well as the PLRP-S column performed significantly better than the PepMap column. For the proteins Cyt C and Myo, separation on the PepMap column resulted in broad peaks (exemplary chromatograms for all three columns can be found in Fig. S1 in the SI). Compared to the CoAnn and PLRP-S column, the PepMap contains particles with a small pore size (100 Å for PepMap vs. 1000 Å for CoAnn and PLRP-S). Due to the increased diffusion to the interior of the particles, larger pores are beneficial for intact protein separation [39]. The CoAnn column shows the overall best separation efficiency and resolution which is most likely due to its length of 50 cm. This capillary length, however, increases backpressure and restricts the maximum applicable flow rate to 250 nL/min. Therefore, gradient elution and equilibration are slow, resulting in an overall method length of 90 min. The PLRP-S column delivers an acceptable separation, but the *N* and *R*_S_ values for RNAse A, Cyt C and Lys are considerably lower compared to the CoAnn column. However, due to the low backpressure, the column can be operated at flow rates up to 500 nL/min, allowing fast equilibration of the column. This allows separation and equilibration in 60 instead of 90 min.

**Table 1:** Separation efficiency (*N*) and resolution (*R*) of the model proteins on the three analysed columns. Values were obtained after independent optimisation of separation gradients for the columns respectively. For each value, the standard deviation (*n* = 3) is given

		PepMap		CoAnn		PLRP-S	
*N*	RNAse A (1)	34,259	± 2038	200,355	± 19,092	85,107	± 3771
	Cyt C (2)	4530	± 360	142,989	± 19,707	102,216	± 688
	Lys (3)	46,284	± 1355	172,372	± 25,874	109,153	± 12,815
	Myo (4)	17,297	± 1385	205,546	± 13,273	371,286	± 2215
*R*_S_^a^	1–2	3.6	± 0.1	13.6	± 0.9	9.6	± 0.2
	2–3	3.2	± 0.1	8.0	± 0.6	5.5	± 0.1
	3–4	7.1	± 0.2	19.9	± 1.0	8.8	± 0.2
*t*^b^		60 min		90 min		60 min	

^a^Resolution between the peak numbers as mentioned in the corresponding row

^b^Total method run time

Based on the aforementioned results, both columns, the CoAnn Tech as well as the PLRP-S column, are considered suitable for the 2D platform. If high separation efficiency and maximal peak capacity are needed, for example, for the analysis of complex biological samples, the CoAnn Tech is the column of choice. The PLRP-S column on the other hand allows for a higher throughput while providing sufficient separation performance. In the context of this study, we selected the PLRP-S column for the nanoLC-CZE-MS platform.

**Fig. 1:**
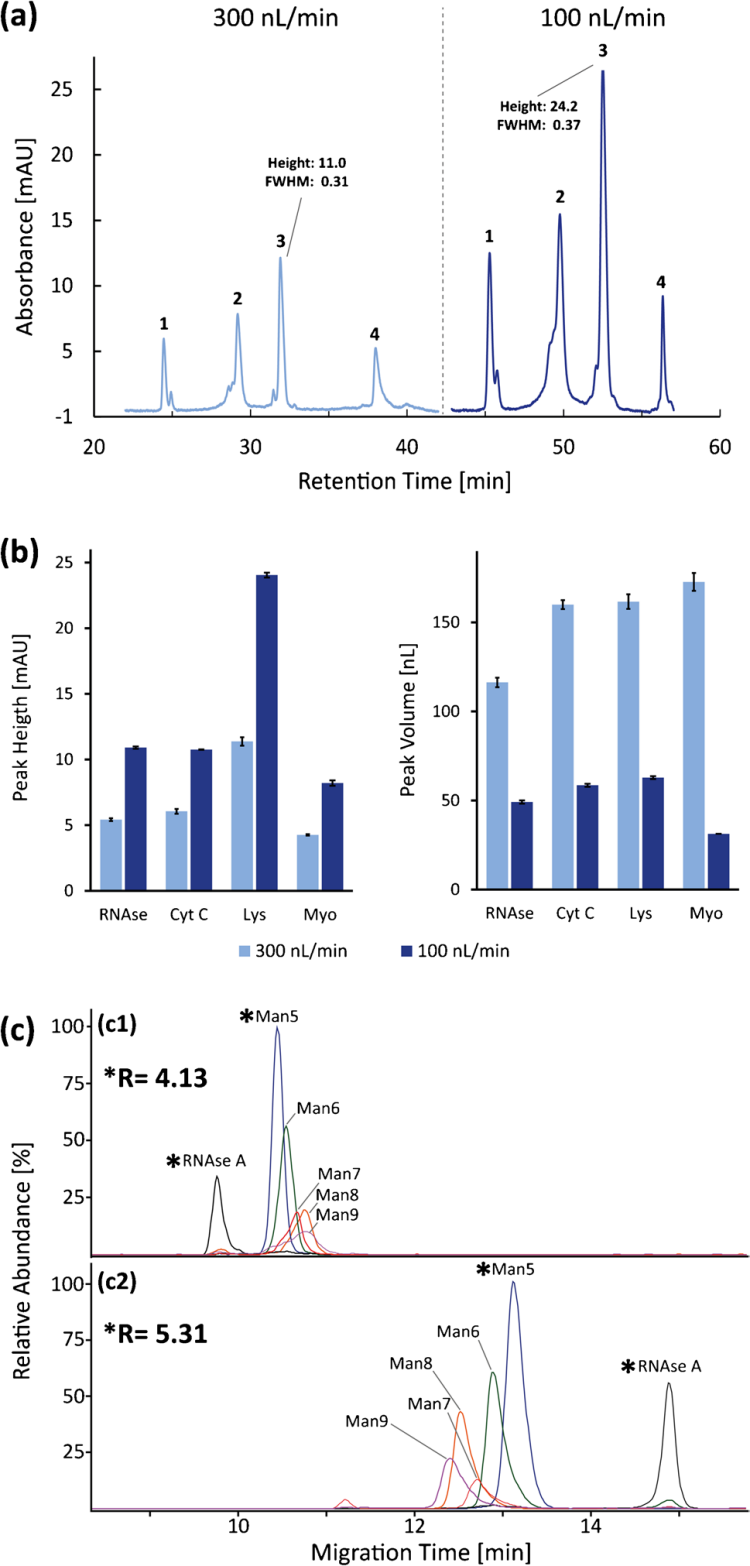
Optimization of nanoLC and CZE parameters for the 2D setup. (**a**) Representative chromatogram of 6 μg/mL protein mixture (1 μL injected) with 300 and 100 nL/min flow rate, respectively, on the PLRP-S column. 1: RNAse A, 2: Cyt C, 3: Lys, 4: Myo. (**b**) Peak height and peak volume for the four proteins for 300 and 100 nL/min flow rate, respectively. Error bars: standard deviation (*n* = 3). (**c**) EIEs of RNAse A and RNAse B glycoforms by CZE-MS. (c1) 70-cm PVA-coated capillary, (c2) 70-cm PDADMAC-PMA-coated capillary. 0.2 M FA BGE, respectively. The resolution between RNAse A and the Man 5 glycoform is given in the respective electropherogram. The according peaks are marked with an asterisk

For the nanoLC method with 300 nL/min flow rate, the volume of a peak with a FWHM of 0.35 min is approximately 200 nL. For a transfer volume of 20 nL (highest loop volume of the valve used in this study), this corresponds to a transfer efficiency of 10% (volume) and 18% (analyte) respectively. The transfer efficiency describes the fraction of the ^1^D peak that is transferred to the ^2^D and is described in terms of either the total peak volume or the total analyte amount. When using the total analyte amount, the Gaussian peak shape is considered to calculate the transfer efficiency. To increase the transfer efficiency and hence the sensitivity of the 2D platform, the flow rate of the nanoLC dimension was reduced to 100 nL/min. Fig. 1(a) shows chromatograms for 300 nL/min and 100 nL/min respectively (same concentration and injection volume). While retention times increased at the lower flow rate, peak widths increased only slightly. In the case of myoglobin, the peak width even decreased with decreasing flow rate. Myoglobin possesses a low diffusion coefficient of 0.66*10^−8^ m^2^ V^−1^ s^−1^ [8], and thus, it is plausible that mass transfer is enhanced at lower flow rates. Fig. 1(b) shows the effect of the reduced flow rate on peak heights and volumes. Peak heights increase with lower flow rates due to the decreased dilution of the injected sample. Peak volumes decrease by factors between ~ 2.5 and ~ 5.5. Peak volumes of ~ 60 nL increase the transfer efficiency to 33% (volume) and 55% (analyte). This transfer efficiency is higher than in our previous report [21] and considerably higher than typical transfer efficiencies of ~ 1% that can be achieved with flow-gating interfaces [40]. We consider this transfer efficiency of 55% (analyte) as an optimum as a further increase in transfer efficiency increases the likelihood of co-transferring adjacent peaks due to a transfer window that is too wide.

Depending on the application, it is possible to freely switch between the LC method with 300 nL/min (faster separation) and 100 nL/min (higher transfer efficiency). Both flow rates were used in the following experiments depending on the respective application.

### CZE conditions

Capillary coatings are vital to reduce undesired interactions between the analytes and the capillary wall for the separation of intact proteins [41]. In our previous proof-of-concept study, we used PVA-coated capillaries for the ^2^D. Although being effective in reducing protein interactions, some problems with the PVA coating remain: (i) The coating procedure is complex and time-consuming, (ii) batch-to-batch variations tend to be high, and (iii) the lack of an electroosmotic flow (EOF) makes the system very susceptible to pressure fluctuations and current instabilities. This is especially true for the application in the 2D setup where the separation is performed through the four-port valve.

Successive multiple ionic-polymer layer (SMIL) coatings offer a high separation efficiency based on a reversed EOF (5 layers, 5^th^ layer cationic), while the coating protocols are easy, quick and reproducible [42–44]. We compared the performance of a PVA and a 5-layer PDADMAC-PMA-coated capillary (70 cm length respectively) for the separation of RNAse B glycoforms. Fig. 1(c) shows extracted ion electropherograms for both coatings. Due to the reversed EOF on the PDADMAC-PMA-coated capillary, the migration order is reversed compared to the PVA coating. Separation takes slightly longer on the PDADMAC-PMA-coated capillary while the overall separation of the glycoforms is enhanced as representatively demonstrated by the resolution between RNAse A and glycoform Man 5 (5.31 for PDADMAC-PMA vs. 4.31 for PVA).

The high separation performance of SMIL coatings combined with an easy, quick and reproducible coating protocol makes these types of coating ideal for application in the 2D platform. Furthermore, in our experience, the presence of a high EOF proved to be beneficial for the analytical stability and reproducibility of the overall 2D setup. Detailed information on capillary preparation, coating stability and storage can be found elsewhere [42, 43, 45].

### Setup of the nanoLC-CZE-MS platform

**Fig. 2:**
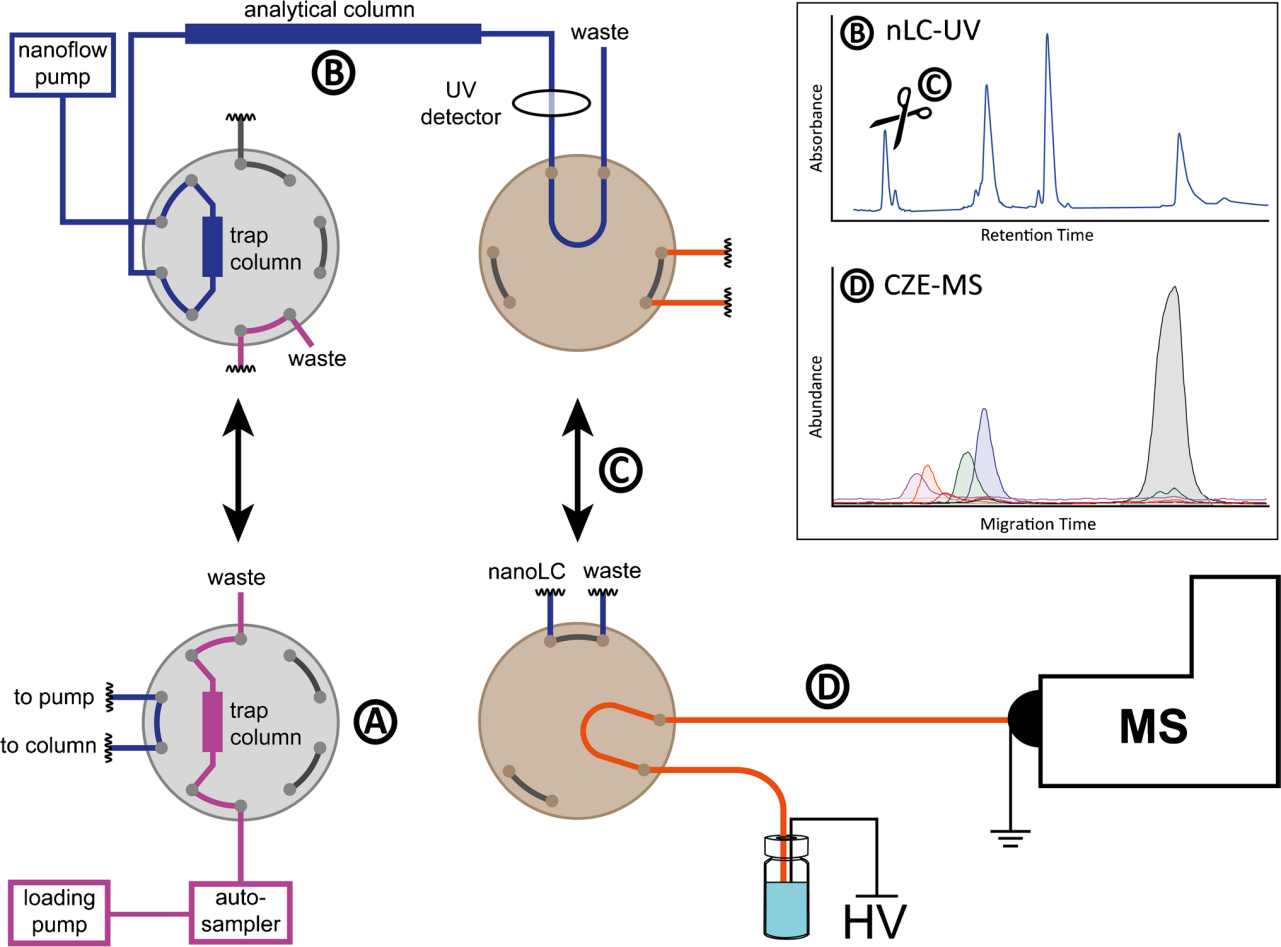
Fluidic setup of the nanoLC-CZE-MS platform with A–D highlighting the subsequent steps in analysis. (**A**) The sample is loaded on the trap column via the loading pump. (**B**) The separation is performed via the nanoflow pump over the analytical column. The column is connected to a 30-μm ID capillary with a UV window for detection via an external UV detector. The UV window is placed ~ 12 cm prior to the valve entrance. The LC effluent is carried through the four-port valve equipped with a rotor (20-nL internal loop volume). (**C**) When the peak of interest reaches the detector, the valve is switched after the appropriate delay time. (**D**) The transferred fraction is analysed by CZE-MS. The box on the top right corner shows a schematic chromatogram of the ^1^D separation and an electropherogram of the ^2^D according to the transferred fraction

After independent optimization of the LC and CZE dimensions, they were combined in the 2D setup (Fig. 2). The ^1^D separation was performed with either of the two nanoLC methods (300 or 100 nL/min) as described before. To couple the ^1^D with the ^2^D, a mechanical four-port valve with an internal 20-nL loop on the rotor was used as previously described in detail [18, 21]. The detection capillary of the nanoLC was connected to one of the ports of the valve with ~ 12 cm between the UV window and valve inlet. The nanoLC effluent is carried through the valve to the waste (Fig. 2(B)). Upon detection of the peak in the UV chromatogram, the valve was switched after the appropriate delay time, as calculated by using the capillary dimensions and the nanoLC flow rate. Switching of the valve (Fig. 2(C)) allows a fraction of the peak to be transferred to the ^2^D. For the ^2^D, the inlet capillary (35 cm) was connected to the four-port valve. The other free port of the valve was connected to the separation capillary (70 cm).

One major and critical characteristic of the nanoLC-CZE-MS platform is the “cut precision”. The peak of interest is transferred to the ^2^D by calculating the time a fraction needs to traverse from the UV window to the loop of the valve. To characterise small or narrow peaks, or to selectively transfer peaks from a complex sample, the transfer procedure has to be precise. Therefore, we evaluated the precision in cutting by multiple transfer of Lys from the four-protein model mixture (20 μg/mL). We decided to use Lys because the protein exhibits only a single proteoform, allowing straight evaluation of precision based on the peak area of its EIE in CZE-MS. The relative standard deviation (RSD) of the peak areas was 22% (*n* = 6, intra-day repeatability) demonstrating a high precision in transferring the peak apex. Migration times in the combined nanoLC-CZE-MS setup were also repeatable with 1.1% (*n* = 6) intra- and 2.3% (*n* = 12) inter-day RSD (3 days, 6 + 3 + 3 measurements). The raw data for this calculation can be found in Tables S2 and S3 in the SI. Besides the good migration time stability, the application of PDADMAC-PMA-coated capillaries additionally increased the ruggedness of the setup in terms of current stability compared to the previously used PVA coating. As mentioned before, we attribute this increased analytical stability to the presence of an EOF in the CE dimension, making the CZE dimension less susceptible to syphoning effects, slight pressure gradients and small air bubbles.

### Sensitivity of the nanoLC-CZE-MS platform

The substantially higher loadability of the nanoLC is expected to result in a higher concentration sensitivity of the nanoLC-CZE-MS platform compared to CZE-MS alone. To evaluate this capability, the protein mix was prepared in different dilutions to create calibration curves with CZE-MS and nanoLC-CZE-MS. For the calibration of the CZE-MS platform, concentrations of 100, 80, 60, 40 and 20 μg/mL with an injection volume of 20 nL were used. For the nanoLC-CZE-MS platform, 5, 3, 1 and 0.5 μg/mL with an injection volume of 20 μL on the nanoLC were used. For nanoLC-CZE-MS, Lys was transferred from ^1^D to ^2^D.

**Fig. 3:**
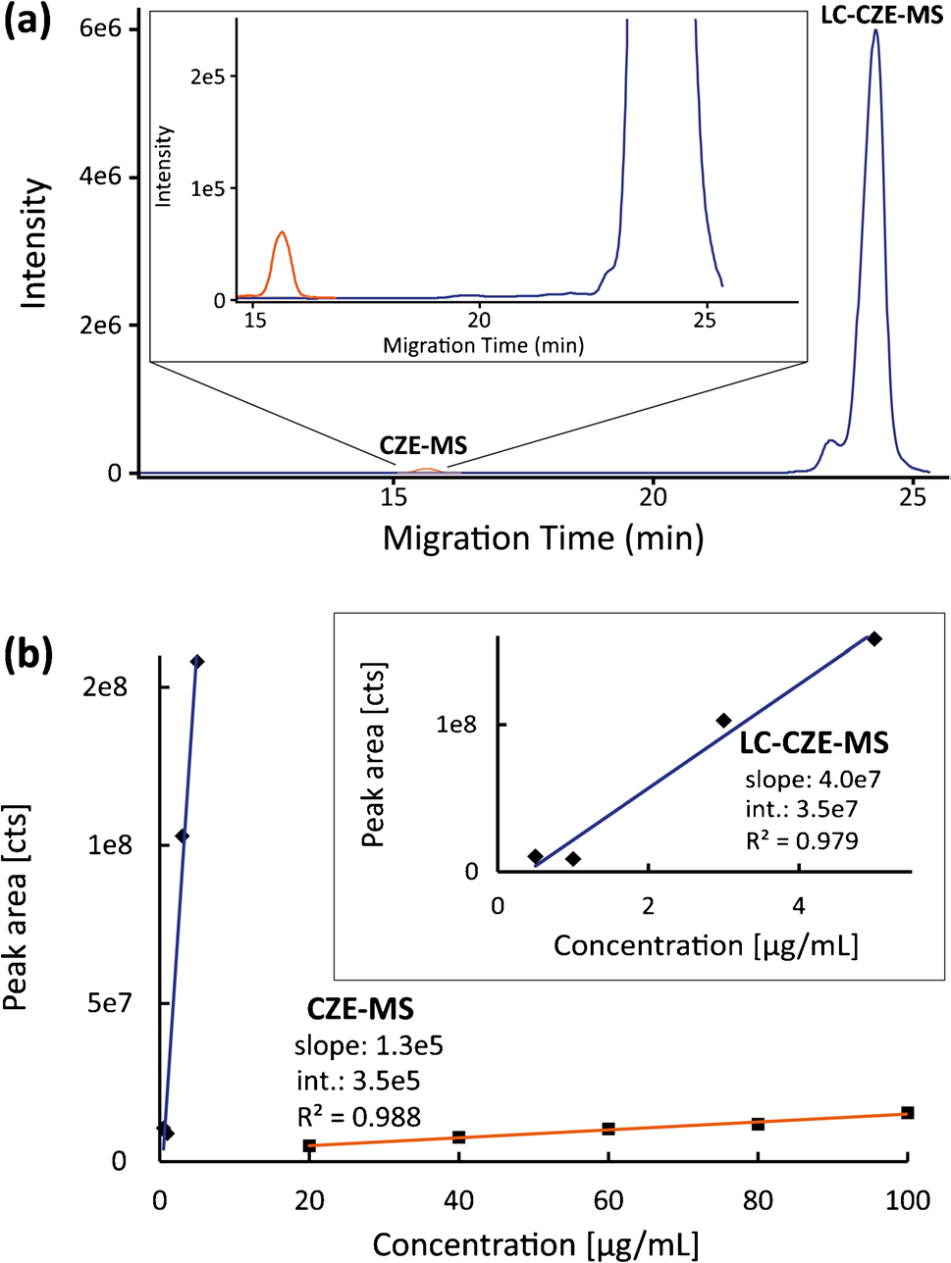
Concentration sensitivity of CZE-MS vs. nanoLC-CZE-MS. (**a**) Representative EIEs of lysozyme (5 μg/mL) analysed by CZE-MS and nanoLC-CZE-MS. (**b**) Calibration curves for lysozyme by CZE-MS and nanoLC-CZE-MS respectively

As expected, peak areas for Lys were considerably higher in nanoLC-CZE-MS compared to those in CZE-MS (Fig. 3(a)). To quantify the differences in concentration sensitivity, the slopes of the calibration curves for Lys were used. Fig. 3(b) shows the calibration curves for the two instrumental setups. The nanoLC-CZE-MS approach exhibits a 280-fold higher slope, hence concentration sensitivity for Lys compared to CZE-MS. As separation and MS conditions in the ^2^D are the same as in the CZE-MS method, we assume the higher loadability to be the sole reason for this increased sensitivity. The obtained factor is slightly lower than the maximum possible improvement factor of 500 (1000-fold higher injection volume and ~ 50% transfer efficiency of the analyte). We expect similar factors are achievable for other analytes and samples.

Interestingly, the limiting factor towards analysing concentrations < 0.5 μg/mL was neither the UV nor the MS sensitivity but protein adsorption phenomena that were present at such low concentrations. To characterise very-low-concentrated protein samples, additional strategies to reduce adsorption during sample preparation and on sample vials are necessary.

### Analysis of alpha-1-acid glycoforms by nanoLC-CZE-MS

To fully harness the potential of such a 2D separation platform, we analysed human AGP. AGP is present in three sequence variants: F1 (wild type), S (Q38 ➔ R) and F2 (V174 ➔ M). Furthermore, it is heavily glycosylated and exhibits 5 glycosylation sites [28]. On each of the glycosylation sites, the glycan structures vary in the number of antennas, fucosylation and sialyation. Analysis of the glycosylation profile of AGP promises an interesting clinical approach as altered glycosylation has been linked to several disease conditions [31–33]. In a previous study, it was shown that CZE-MS allows detailed characterisation of AGP glycoforms on intact level [29].

**Fig. 4:**
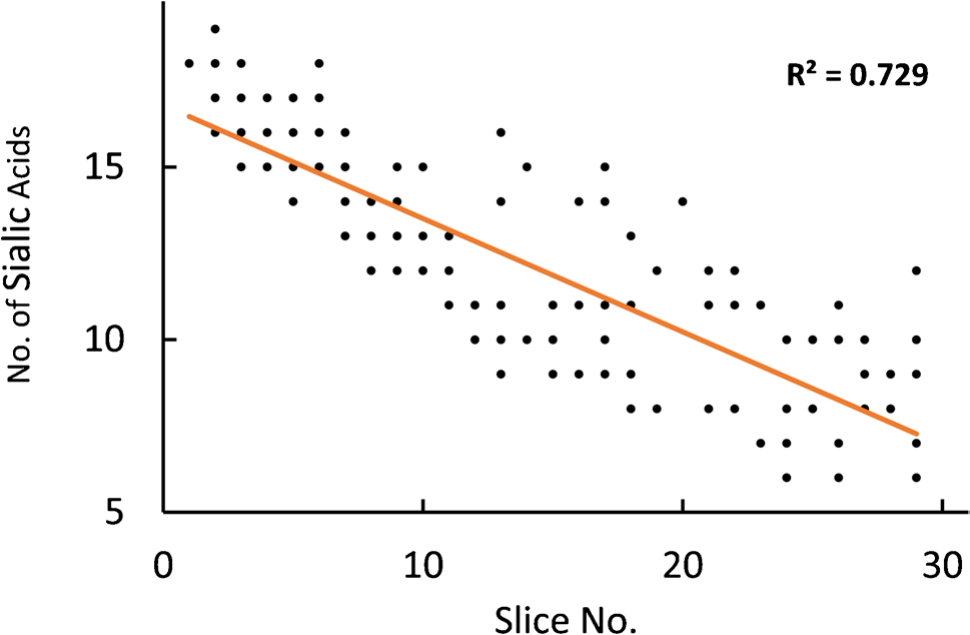
Number of sialic acids vs. slice number of assigned glycoforms by CZE-MS. The orange line represents a linear regression function to show correlation

As a benchmark for comparison, we performed the glycosylation profiling of a 1-mg/mL solution AGP with CZE-MS first. As the high content of sialic acids on the protein results in a low pI (2.8–3.8 [28]), hence low mobility in CZE, we decided to adapt the CZE-MS separation system. By using a SMIL coating with a lower EOF (DEAEDq-PMA), resolution can be enhanced for slowly migrating species [37]. CZE-MS resulted in a broad peak of overlapping glycoforms (Fig. S2). For putative glycoform assignment, 30-s time slices were created to average mass spectra and perform subsequent mass deconvolution. The intact masses of all slices were matched against the theoretical masses of the glycoforms for assignment. One hundred eighty-six glycoforms of AGP were detected of which 92 could be attributed to the F1, 74 to the S and 20 to the F2 sequence variant. This number is comparable to the number previously reported [29]. Fig. 4 shows a plot of the number of sialic acids against the number of the respective time slice in CZE-MS. The correlation indicates that the sialyation of the proteoforms is the main aspect for selectivity in the CZE dimension as it effectively changes the charge of the protein in the solution.

**Fig. 5:**
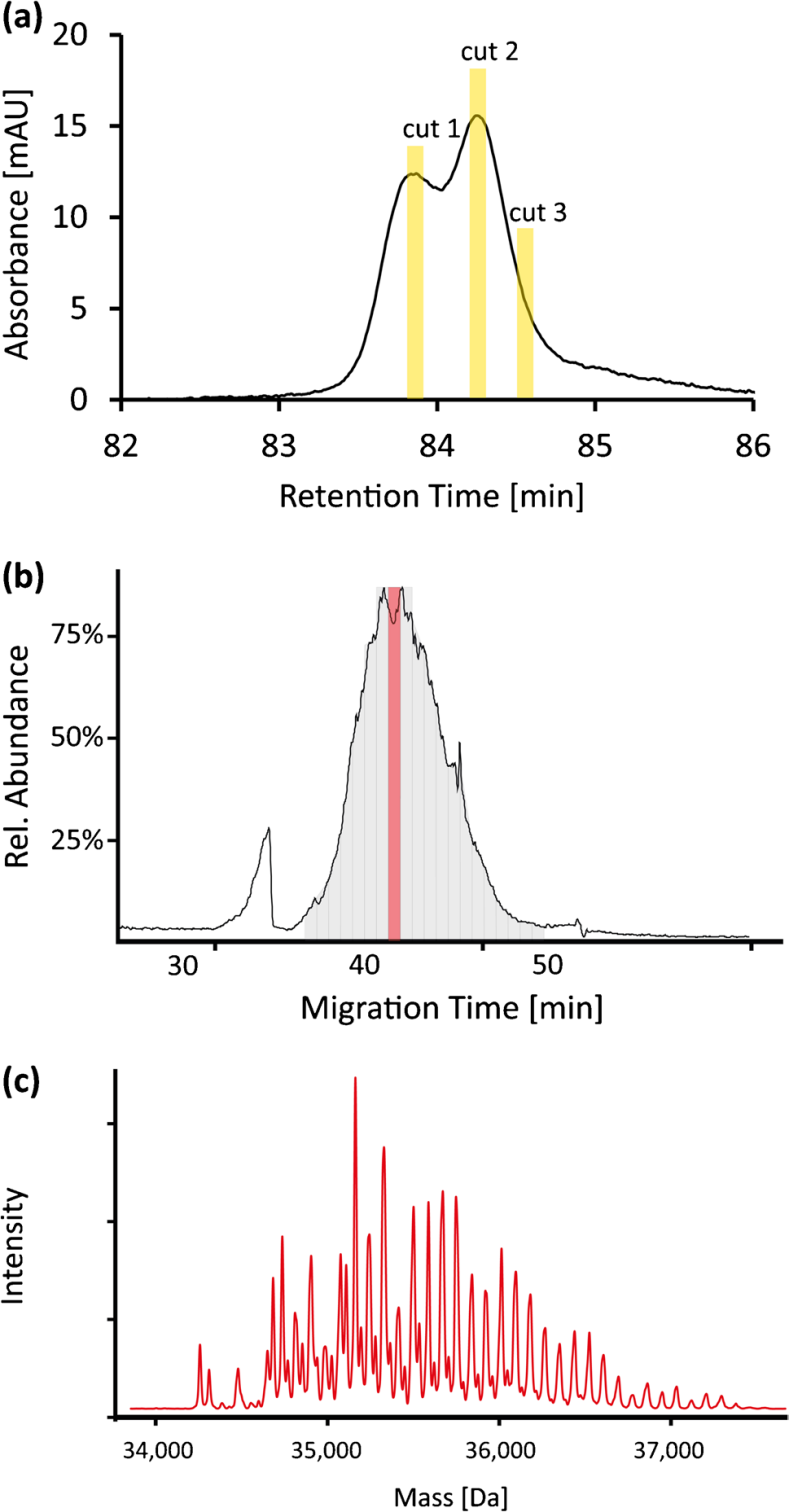
General principle of AGP characterisation by nanoLC-CZE-MS. (**a**) NanoLC separation of human AGP (50 μg/mL, 10 μL injection) including the three parts of the double peak that were transferred to the ^2^D. (**b**) BPE of cut 2. To assign AGP glycoforms, 30-s time slices were created across the broad, unresolved peak. (**c**) Deconvoluted mass spectra of the marked time slice

Despite the good selectivity of the CZE separation based on the sialyation of the protein, a single dimension is not sufficient to adequately separate this highly complex mixture of proteoforms. We evaluated the use of nanoLC-CZE-MS for glycosylation profiling of AGP and compared it to CZE-MS. For the application of nanoLC-CZE-MS, the following benefits could be expected: (i) The increased sensitivity allows AGP glycosylation profiling from lower-concentrated samples. (ii) The nanoLC separation enables separation of the matrix or other proteins (if present), and (iii) the LC dimension allows a pre-separation of AGP proteoforms, reducing complexity in the CZE-MS dimension, enabling detection of low-abundant proteoforms. Fig. 5 shows the general principle of AGP glycoform profiling by nanoLC-CZE-MS. NanoLC separation of AGP results in a double peak (Fig. 5(a)). From this peak, three individual fractions were transferred to the ^2^D, resulting in the separation of various glycoforms in the ^2^D (Fig. 5(b)). Time slicing and deconvolution were performed as described for CZE-MS (Fig. 5(c)). By combining three cuts, 368 putative glycoforms could be assigned by nanoLC-CZE-MS from a 50-μg/mL sample. This represents roughly twice as many putative glycoforms from a 20-fold lower-concentrated sample compared to CZE-MS.

**Fig. 6:**
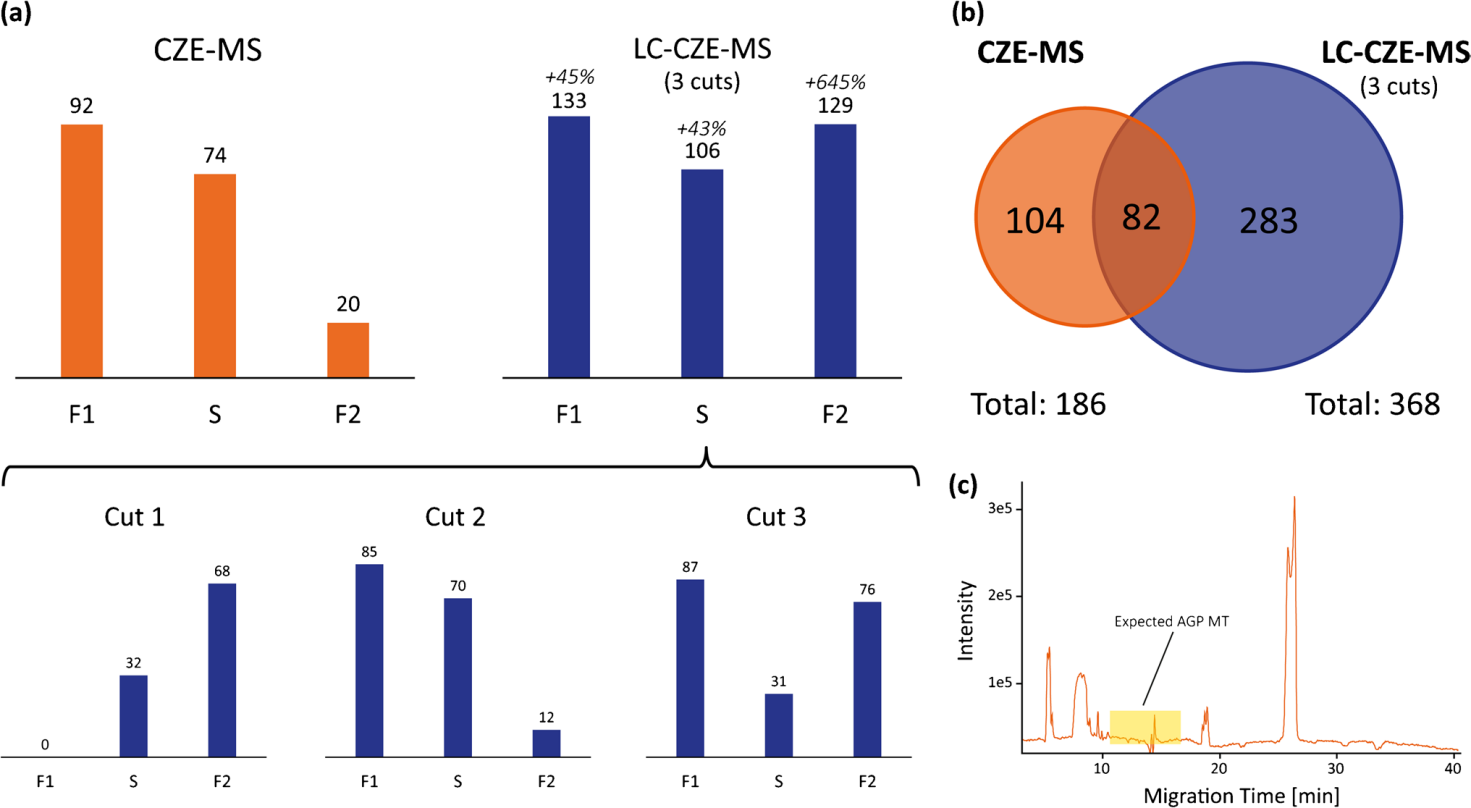
Number of assigned AGP glycoforms in CZE-MS and nanoLC-CZE-MS. (**a**) Number of assigned glycoforms by sequence variant by CZE-MS and nanoLC-CZE-MS by the combination of three individual cuts from the ^1^D. In the lower panel, the assignments for the individual cuts can be seen. (**b**) Venn diagram comparing the assigned glycoforms in CZE-MS and nanoLC-CZE-MS. (**c**) CZE-MS of 25 μL/min AGP spiked to 1:20 diluted DBS eluate. In the expected MT window for AGP, no signals were detected

Fig. 6(a) shows the number of assigned putative glycoforms for the three major sequence variants between CZE-MS and nanoLC-CZE-MS. Combining all three cuts, 133 (F1), 106 (S) and 129 (F2) glycoforms were assigned. Compared to the CZE-MS approach, this represents an increase of 45%, 43% and 645% respectively. The high increase for the F2 sequence variant was especially surprising. The relative intensities for each assigned glycoform were determined after deconvolution and normalised to the intensity of the most abundant glycoform for each of the cuts. Comparing these normalised intensities for the three variants, it can be seen that the median relative intensity for F2 is lower than for F1 and S (9.8% for F2 vs. 11.9% and 16.0% for F1 and S respectively). A corresponding table and histogram can be found in the SI. We suspect that the lower abundance of F2 glycoforms could be the main reason for the significantly higher assignment rates with the nanoLC-CZE-MS approach. As the nanoLC-CZE-MS approach reduces the complexity of the protein mix that is simultaneously introduced in the MS, low-abundant species can be identified more confidently due to the reduction of ion suppression effects.

Interestingly, the F1 variant was only detected in cuts 2 and 3. The highest number of putative glycoforms for the S variant was detected in cut 2. For F2, 68 and 76 glycoforms could be assigned in cut 1 and 3 while only 12 were assigned in cut 2. This suggests that chromatographic separation is partially driven by the presence of the different sequence variants. Interestingly, however, the behaviour of the F2 variant suggests that this process is not a simple separation of the three sequence variants, but it appears to be a more complex separation principle combining effects comprising sequence variant and glycosylation.

Fig. 6(b) shows a Venn diagram comparing the number of putative glycoforms between CZE-MS and nanoLC-CZE-MS. The number of putative glycoforms that were assigned by both of the techniques is 82. One hundred four unique glycoforms could be assigned by CZE-MS alone. This can be explained by the incomplete transfer of fractions along the LC double peak from the ^1^D to the ^2^D. We suspect the proportion of uniquely assigned putative glycoforms in CZE-MS to decrease when more cuts from the ^1^D are transferred to the ^2^D. At the same time, this would increase both the commonly assigned as well as the uniquely assigned putative glycoforms for nanoLC-CZE-MS.

As previously mentioned, AGP is an important plasma protein and a potential biomarker for various diseases. To evaluate the general applicability of the nanoLC-CZE-MS platform for AGP glycosylation profiling in clinical samples, we spiked 25 μg/mL AGP to 1:20 diluted DBS eluate. We chose DBS as a model matrix as samples were readily available and contain high abundant matrix proteins (mainly haemoglobin). Before analysing the spiked samples, we evaluated whether the DBS eluate contains endogenous AGP. In nanoLC analysis, we did not observe any peak for AGP. Presumably, the stability of AGP on the DBS is not sufficient to extract it in relevant concentrations. The spiked DBS eluate samples were then analysed with the same cutting scheme as shown in Fig. 5(a). As can be seen in Fig. S3 in the SI, besides AGP, haemoglobin was identified as a prominent peak. It is separated from AGP and does not hamper AGP glycoform profiling in the ^2^D. Overall, 229 glycoforms were assigned which is 37% less than assigned for the AGP solved in UPW. This reduced number of assigned glycoforms is presumably due to a combination of the lower concentration used in this experiment and the complex sample matrix. Typical AGP concentrations in human plasma are between 600 and 1200 μg/mL [46]. Considering a sample preparation with sample loss and dilution (e.g. the 1:20 dilution used here for the DBS eluate), a final concentration of 25 μg/mL lies within the range to be obtained from clinical samples. Fig. 6(c) shows the base peak electropherogram of a CZE-MS separation (1D) of the spiked DBS eluate. Only the haemoglobin peak as well as some other smaller peaks are visible in the electropherogram. In the time window of AGP, no signal could be obtained. The concentration of 25 μg/mL AGP is too low to be detected in CZE-MS which again demonstrates the value of the nanoLC-CZE-MS in preconcentration. We want to emphasise here that we did not evaluate the usage of DBS for AGP analysis in the clinical context but rather wanted to show the general applicability of the platform to complex samples.

In conclusion, by combining high sensitivity and different selectivities, the nanoLC-CZE-MS platform enables AGP characterisation in physiologically relevant concentrations from complex biological matrices which is not directly possible by CZE-MS alone.

## Concluding remarks

Here, we report a heart-cut nanoLC-CZE-MS platform with optimised separation conditions for intact proteins, high transfer efficiency between the two dimensions and high sensitivity. To our knowledge, this is the first well-described LC-CZE-MS platform for the analysis of intact proteins. A polymer-based stationary phase (PLRP-S) in the ^1^D combined with SMIL-coated capillaries in the ^2^D provides favourable conditions for intact protein separation. The type of SMIL coating can be adapted to the mobility of the protein to maximise separation capabilities for different protein groups. With the optimised operational conditions, an ideal transfer efficiency of around 50% was achieved which is considerably higher than what has been reported so far. Furthermore, repeatability of the valve-based transfer was demonstrated. The combination of high transfer efficiency and considerably increased loadability of nanoLC compared to CZE results in high sensitivity. The nanoLC-CZE-MS platform exhibits 2–3 orders of magnitude higher sensitivity compared to CZE-MS alone.

NanoLC-CZE-MS was used to characterise the glycosylation profile of human AGP. By transferring three fractions from the ^1^D to the ^2^D, the number of assigned glycoforms could be doubled using a 20-fold lower-concentrated sample compared to CZE-MS alone. This underlines the combined benefits of the high sensitivity and the orthogonality of the separation dimensions. In fact, the two separation dimensions address different molecular characteristics of the proteins. The hydrophobicity-based LC separation separates different proteins based on their different amino acid sequences. The mobility-based CZE separation on the other hand has a high selectivity for PTMs that change the overall charge of the proteoform. This principle was demonstrated by the analysis of AGP spiked to the DBS eluate. The nanoLC dimension allowed efficient separation of matrix proteins. The CZE-MS analysis enabled the separation and detection of various AGP glycoforms.

To further exploit the capabilities of nanoLC-CZE-MS, we aspire to further optimise our setup by the implementation of multiple heart-cut or selective comprehensive mode and extending the number of peaks to be transferred by using multi-segment injections in the ^2^D. This will enable even more detailed characterisation of complex biological samples on the level of intact proteins and their respective proteoforms.

## Supplementary Information

ESM 1 (DOCX 604 kb)

## Data Availability

Not applicable
