# Supplementary material for: Characterisation of a new online nanoLC-CZE-MS platform and application for the glycosylation profiling of alpha-1-acid glycoprotein
Source: Anal Bioanal Chem. 2021 Dec 9;414(5):1745–57. doi: 10.1007/s00216-021-03814-6 (PMC8791864; doi:10.1007/s00216-021-03814-6)
Supplement: Supplementary file 1 — (DOCX 604 kb) [file 216_2021_3814_MOESM1_ESM.docx]

Supplementary Information

Characterisation of a New Online NanoLC-CZE-MS Platform and Application for the Glycosylation Profiling of Alpha-1-Acid Glycoprotein

Alexander Stolz^1,2^, Christian Neusüß^1^*

^1^Faculty of Chemistry, Aalen University, Aalen, Germany

^2^Department of Pharmaceutical and Medicinal Chemistry, Friedrich Schiller University, 07743 Jena, Germany

***Correspondence:** Prof. Dr. Christian Neusüß, Beethovenstr. 1, 73430 Aalen, Germany, Christian.Neusuess@hs-aalen.de

# Table of Contents

1. Additional Methodological Information
2. m/z List used to create EIEs
3. Column Comparison
4. CZE-MS of AGP
5. Relative Intensities of Assigned Glycoforms
6. NanoLC of DBS Eluate Spiked With AGP
7. Assigned Glycoforms by CZE-MS and nanoLC-CZE-MS

# Additional Methodological Information

## nanoLC methods for all columns

For all methods without a trap column, 1 µL sample was directly loaded on the analytical column.

### PepMap

The gradient (300 nL/min) started for 4 min at 15% B, followed by a linear gradient to 70% B for 38 min. The column was flushed for 5 min with 95% B before column equilibration at 15% B for 18 min.Overall method length: 60 min

### CoAnn:

The gradient (250 nL/min) started for 4 min at 15% B, followed by a linear gradient to 90% B for 60 min. The column was equilibrated at 15% B for 30 min. Overall method length: 90 min.

### PLRP-S, 300 nL/min, on column

The gradient for the 300 nL/min method started at 15% B for 4 minutes followed by a 31 min linear gradient to 60% B. The column was flushed with 95% B for 10 minutes before equilibrating at 15% B for 20 min. Overall method length: 60 min

### PLRP-S, 100 nL/min, on column

The 100 nL/min method started with 4 min at 15% B with a flow rate of 500 nL/min followed by a linear gradient to 60% B in 31 min with 100 nL/min. The column was flushed at 95% B for 10 minutes followed by column equilibration for 20 min. Finally, the flow rate was increased to 500 nL/min at 15% B for another 5 min. Overall method length: 70 min

### PLRP-S, 300 nL/min, preconc.

To allow the injection of higher sample volumes (up to 20 µL) a 0.15 x 50-60 mm trap column (C4, 3 µm, 300 Å) was used. The trap column was loaded with 15% B at a flow rate of 4 µL/min for 10 min while the analytical column was equilibrated with 500 nL/min and 15% B. After loading the column switching valve was switched and separation was performed with a linear gradient of 32 min to 60% B at 300 nL/min flow rate. The column was flushed with 95% B for 7 minutes before re-equilibration at 15% B for 10 min. Overall method length: 60 min

### PLRP-S, 100 nL/min, preconc.

For the 100 nL/min method, loading was performed the same way while column equilibration was performed at 500 nL/min. After switching the valve, separation was performed with a linear gradient of 31 min to 60% B, followed by 17 min column flushing at 95% B at 100 nL/min. Column equilibration was performed at 15% B for 10 min at 100 nL/min and another 5 minutes at 500 nL/min. Overall method length: 75 min

## MatLab script for baseline correction

Due to the change of refractive index of the nanoLC effluent during gradient elution, a strong but repeatable baseline drift occurs. To correct the chromatogram from this baseline drift an automated MatLab (R2017b, Mathworks, Natick, USA) script was written. For the baseline correction, the UV signal of a blank run is used to perform a second-order polynomial regression which accurately describes the baseline drift (R^2^>0.99). The resulting function is subtracted from the x-y dateset of the chromatogram of interest to obtain the baseline-corrected x-y dataset.

# *m/z* List used to create EIEs

Table S1. *m/z* values used to create EIEs for the respective proteins. For each EIE, the intensities of the three most abundant charge states for each protein were summed.

| Protein | | *m/z* values used for EIEs | | | | | |
| --- | --- | --- | --- | --- | --- | --- | --- |
|  |  | *m/z 1* | z | *m/z 2* | z | *m/z 3* | z |
| lysozyme | | 1590.4 | 9 | 1789.1 | 8 | 2044.5 | 7 |
| RNAse A | | 1369.2 | 10 | 1521.2 | 9 | 1711.3 | 8 |
| RNAse B | |  |  |  |  |  |  |
|  | Man5 | 1355.4 | 11 | 1490.8 | 10 | 1656.4 | 9 |
|  | Man 6 | 1370.1 | 11 | 1507.1 | 10 | 1674.4 | 9 |
|  | Man 7 | 1384.9 | 11 | 1523.3 | 10 | 1692.4 | 9 |
|  | Man 8 | 1399.6 | 11 | 1539.5 | 10 | 1710.4 | 9 |
|  | Man 9 | 1414.4 | 11 | 1555.8 | 10 | 1728.5 | 9 |

# Column Comparison


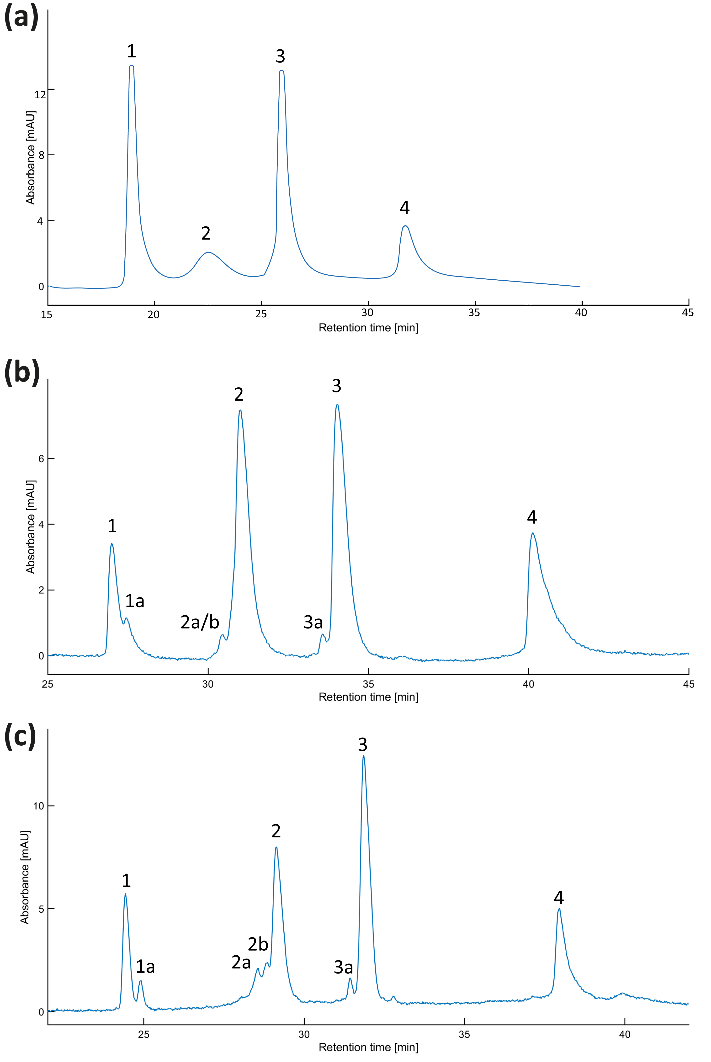


Fig. S1. Separation of the protein mix on three different columns. (a) PepMap C18 column (75 µm x 150 mm), (b) C4 column (75 x 500 mm) and (c) PLRP-S column (75 µm x 250 mm). 1: RNAse A, 2: Cyt C, 3: Lys, 4: Myo. All separations were performed after gradient optimisation.

# Raw Data for Repeatability

Table S2. Raw data for the determination of intra-day repeatability of nanoLC-CZE-MS. For each run, Lys was cut and transferred to the ^2^D.

| Run | Migration Time [min] | Peak Area [cts] |
| --- | --- | --- |
| run 1 | 25.4 | 6.11E+05 |
| run 2 | 25.8 | 3.66E+05 |
| run 3 | 25.2 | 4.69E+05 |
| run 4 | 25.4 | 6.55E+05 |
| run 5 | 25.0 | 6.68E+05 |
| run 6 | 25.5 | 6.57E+05 |
| Mean | 25.4 | 5.71E+05 |
| SD | 0.3 | 1.25E+05 |
| **RSD** | **1.1%** | **21.9%** |

Table S3. Raw data for the determination of inter-day repeatability of nanoLC-CZE-MS. For each run, Lys was cut and transferred to the ^2^D.

|  | Run | Migration Time [min] | Peak Area [cts] |
| --- | --- | --- | --- |
| Day 1 | run 1 | 25.4 | 6.11E+05 |
|  | run 2 | 25.8 | 3.66E+05 |
|  | run 3 | 25.2 | 4.69E+05 |
|  | run 4 | 25.4 | 6.55E+05 |
|  | run 5 | 25.0 | 6.68E+05 |
|  | run 6 | 25.5 | 6.57E+05 |
|  |  |  |  |
| Day 2 | run 1 | 24.9 | 4.77E+05 |
|  | run 2 | 24.9 | 4.91E+05 |
|  | run 3 | 25.2 | 4.91E+05 |
|  |  |  |  |
| Day 3 | run 1 | 24.1 | 2.47E+05 |
|  | run 2 | 23.9 | 2.37E+05 |
|  | run 3 | 24.4 | 2.15E+05 |
|  | Mean | 25.0 | 4.65E+05 |
|  | SD | 0.6 | 1.68E+05 |
|  | **RSD** | **2.3%** | **36.0%** |

# CZE-MS of AGP


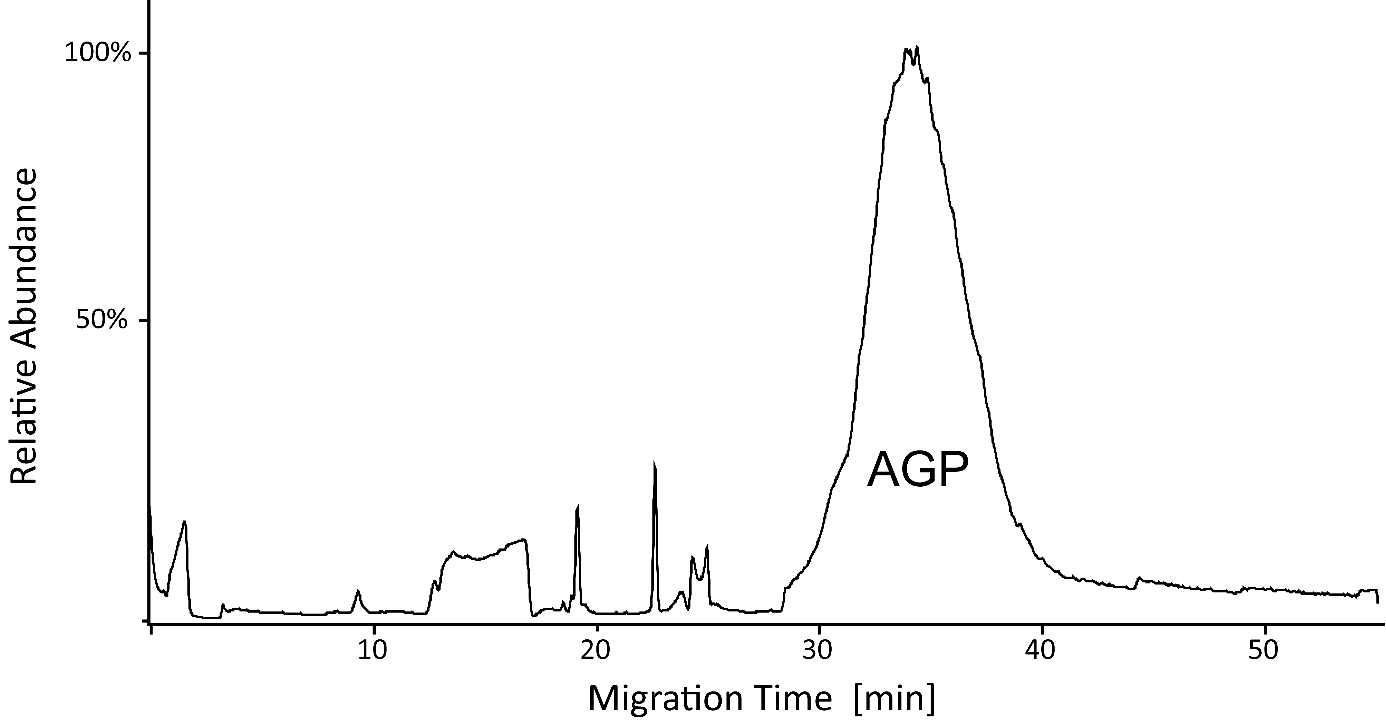


Fig. S2. TIC of AGP (1 mg/mL, 20 nL injection) by CZE-MS. Injection and separation were performed via the valve with DEAEDq-PMA coated capillaries and 2 M HA as BGE. 50% IPA + 1% HA were used as SL. -15 kV separation voltage

# Relative Intensities of Assigned Glycoforms

Table S4. Median, mean and Q1 and Q3 for the relative intensity of all identified glycoforms in LC-CZE-MS.

|  | F1 | S | F2 |
| --- | --- | --- | --- |
| Q1 | 3.0% | 7.3% | 4.5% |
| Median (Q2) | 11.9% | 16.0% | 9.8% |
| Q1 | 23.2% | 26.2% | 18.1% |
| Mean | 16.6% | 18.2% | 12.8% |

# NanoLC of DBS Eluate Spiked With AGP


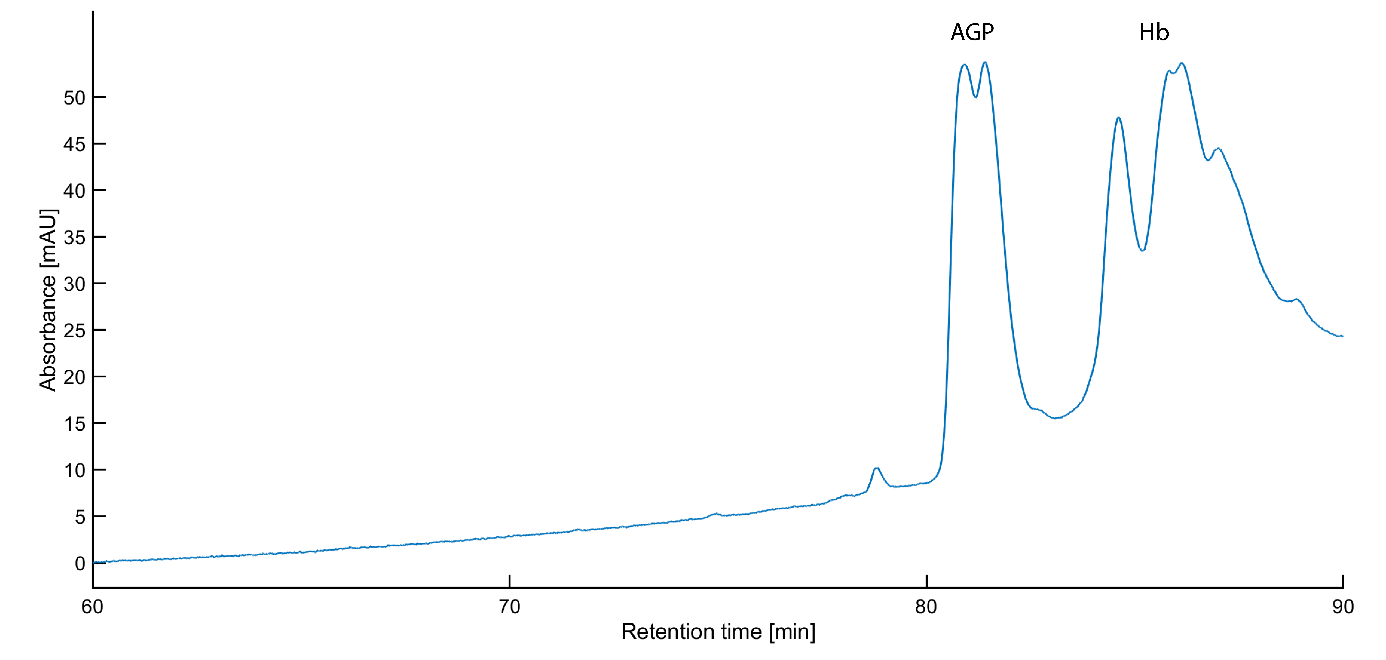


Fig. S3. LC separation of 25 µg/mL AGP spiked in 1:20 diluted DBS eluate (10 µL injection volume). Hb represents the main protein that is present in the DBS eluate.

# Assigned Glycoforms by CZE-MS and nanoLC-CZE-MS

Table S5. Putatively assigned AGP glycoforms by CZE-MS (ordered by decreasing relative intensity).

| Theoretical Mass [Da] | Observed Mass [Da] | Deviation [ppm] | Rel. Int^a^ | Variant | Glycosylation^b^ | | | |
| --- | --- | --- | --- | --- | --- | --- | --- | --- |
|  |  |  |  |  | Hex | HexNac | Fuc | SA |
| 37676.6 | 37676.5 | -3 | 100.00% | F1 | 33 | 28 | 3 | 16 |
| 36217.3 | 36217.4 | 3 | 99.20% | F1 | 31 | 26 | 2 | 14 |
| 35955.1 | 35955.1 | 0 | 90.60% | S | 31 | 26 | 4 | 12 |
| 36145.2 | 36144.9 | -8 | 90.50% | F1 | 32 | 27 | 1 | 13 |
| 37558.5 | 37558.9 | 11 | 87.90% | S | 33 | 28 | 2 | 16 |
| 35560.7 | 35561 | 8 | 86.00% | F1 | 30 | 25 | 2 | 13 |
| 36072.2 | 36072.5 | 8 | 86.00% | F1 | 31 | 26 | 3 | 13 |
| 36436.5 | 36437 | 14 | 83.70% | F1 | 32 | 27 | 1 | 14 |
| 35998.1 | 35998.5 | 11 | 81.90% | F1 | 30 | 25 | 3 | 14 |
| 36144.2 | 36144.5 | 8 | 77.40% | F1 | 30 | 25 | 4 | 14 |
| 36245.3 | 36245.5 | 6 | 76.60% | S | 31 | 26 | 2 | 14 |
| 36536.6 | 36537 | 11 | 75.40% | S | 31 | 26 | 2 | 15 |
| 36364.4 | 36364.5 | 3 | 75.40% | F1 | 33 | 28 | 0 | 13 |
| 35880 | 35880 | 0 | 73.90% | S | 30 | 25 | 2 | 14 |
| 36071.1 | 36071.5 | 11 | 73.40% | F1 | 31 | 26 | 1 | 14 |
| 36610.7 | 36611 | 8 | 72.20% | S | 32 | 27 | 2 | 14 |
| 37384.3 | 37384 | -8 | 70.10% | F1 | 33 | 28 | 1 | 16 |
| 35195.4 | 35195.5 | 3 | 70.10% | F1 | 29 | 24 | 2 | 13 |
| 35853 | 35853.5 | 14 | 64.20% | F1 | 30 | 25 | 4 | 13 |
| 35269.4 | 35269.1 | -9 | 63.80% | F1 | 30 | 25 | 2 | 12 |
| 36435.5 | 36435.5 | 0 | 63.00% | F1 | 30 | 25 | 4 | 15 |
| 34904.1 | 34904.4 | 9 | 59.00% | F1 | 29 | 24 | 2 | 12 |
| 36799.8 | 36799 | -22 | 58.80% | F1 | 31 | 26 | 2 | 16 |
| 36246.4 | 36246.5 | 3 | 57.00% | S | 31 | 26 | 4 | 13 |
| 36465.5 | 36466 | 14 | 55.90% | S | 32 | 27 | 3 | 13 |
| 35486.6 | 35487 | 11 | 55.40% | F1 | 29 | 24 | 2 | 14 |
| 38552.4 | 38552 | -10 | 54.70% | F1 | 35 | 30 | 2 | 17 |
| 37849.8 | 37850 | 5 | 54.10% | S | 33 | 28 | 2 | 17 |
| 36947.9 | 36947.5 | -11 | 53.60% | F1 | 33 | 28 | 2 | 14 |
| 38260.1 | 38260.1 | 0 | 52.60% | F1 | 35 | 30 | 0 | 17 |
| 37704.6 | 37705 | 11 | 51.70% | S | 33 | 28 | 3 | 16 |
| 35589.8 | 35589.9 | 3 | 51.20% | S | 30 | 25 | 4 | 12 |
| 36537.6 | 36537.5 | -3 | 50.10% | S | 31 | 26 | 4 | 14 |
| 37967.8 | 37967.5 | -8 | 49.90% | F1 | 33 | 28 | 3 | 17 |
| 34686.9 | 34686.5 | -12 | 49.80% | F1 | 30 | 25 | 2 | 10 |
| 37457.4 | 37457.5 | 3 | 49.50% | F1 | 32 | 27 | 4 | 16 |
| 36509.6 | 36510 | 11 | 49.30% | F1 | 31 | 26 | 4 | 14 |
| 36538.6 | 36539 | 11 | 47.20% | S | 33 | 28 | 1 | 13 |
| 36391.5 | 36392 | 14 | 47.10% | S | 31 | 26 | 3 | 14 |
| 35445.6 | 35446 | 11 | 44.00% | S | 32 | 27 | 2 | 10 |
| 37456.4 | 37456.5 | 3 | 43.50% | F1 | 32 | 27 | 2 | 17 |
| 35881 | 35881 | 0 | 42.60% | S | 30 | 25 | 4 | 13 |
| 37048.1 | 37048.1 | 0 | 41.60% | S | 32 | 27 | 3 | 15 |
| 37339.3 | 37339.5 | 5 | 41.40% | S | 32 | 27 | 3 | 16 |
| 37238.2 | 37237.5 | -19 | 41.20% | F1 | 33 | 28 | 0 | 16 |
| 36684.7 | 36684.5 | -5 | 41.00% | S | 33 | 28 | 2 | 13 |
| 36975 | 36975.5 | 14 | 40.10% | S | 33 | 28 | 0 | 15 |
| 35735.9 | 35735.5 | -11 | 40.10% | S | 32 | 27 | 0 | 12 |
| 36801.8 | 36801.5 | -8 | 40.10% | F1 | 33 | 28 | 1 | 14 |
| 38187 | 38186.6 | -10 | 40.00% | F1 | 34 | 29 | 2 | 17 |
| 36027.1 | 36027 | -3 | 39.60% | S | 32 | 27 | 0 | 13 |
| 36581.6 | 36581 | -16 | 39.50% | F1 | 32 | 27 | 0 | 15 |
| 35954.1 | 35954 | -3 | 39.30% | S | 31 | 26 | 2 | 13 |
| 37995.9 | 37996 | 3 | 39.30% | S | 33 | 28 | 3 | 17 |
| 36873.9 | 36873.9 | 0 | 38.70% | F1 | 32 | 27 | 2 | 15 |
| 36756.8 | 36756.5 | -8 | 38.50% | S | 32 | 27 | 3 | 14 |
| 35780.9 | 35780.5 | -11 | 37.90% | F1 | 31 | 26 | 3 | 12 |
| 36727.7 | 36727.9 | 5 | 37.10% | F1 | 32 | 27 | 1 | 15 |
| 37894.8 | 37895 | 5 | 37.10% | F1 | 34 | 29 | 0 | 17 |
| 35883 | 35883 | 0 | 36.80% | S | 32 | 27 | 3 | 11 |
| 38989.8 | 38990.5 | 18 | 36.50% | F1 | 35 | 30 | 3 | 18 |
| 38289.2 | 38289 | -5 | 36.40% | S | 35 | 30 | 2 | 16 |
| 36026.1 | 36026.1 | 0 | 36.30% | S | 30 | 25 | 3 | 14 |
| 35268.4 | 35268.6 | 6 | 36.30% | F1 | 30 | 25 | 0 | 13 |
| 37194.2 | 37194.5 | 8 | 35.50% | S | 32 | 27 | 4 | 15 |
| 36508.5 | 36509 | 14 | 35.40% | F1 | 31 | 26 | 2 | 15 |
| 36289.3 | 36289.5 | 6 | 35.40% | F1 | 30 | 25 | 3 | 15 |
| 37268.3 | 37268.5 | 5 | 34.90% | S | 33 | 28 | 4 | 14 |
| 35706.8 | 35707 | 6 | 34.90% | F1 | 30 | 25 | 3 | 13 |
| 35561.7 | 35562 | 8 | 34.80% | F1 | 30 | 25 | 4 | 12 |
| 35196.4 | 35196 | -11 | 34.20% | F1 | 29 | 24 | 4 | 12 |
| 37631.6 | 37632 | 11 | 33.80% | S | 34 | 29 | 0 | 16 |
| 38698.5 | 38698.1 | -10 | 33.60% | F1 | 35 | 30 | 3 | 17 |
| 35051.2 | 35051 | -6 | 33.20% | F1 | 31 | 26 | 0 | 11 |
| 35809.9 | 35809.5 | -11 | 32.50% | S | 33 | 28 | 0 | 11 |
| 36392.5 | 36392.1 | -11 | 32.40% | S | 33 | 28 | 0 | 13 |
| 35372.5 | 35372.1 | -11 | 32.10% | F2 | 29 | 24 | 1 | 14 |
| 38406.2 | 38406.5 | 8 | 31.20% | F1 | 35 | 30 | 1 | 17 |
| 38114 | 38114 | 0 | 31.10% | F1 | 33 | 28 | 4 | 17 |
| 36174.3 | 36174.4 | 3 | 29.70% | S | 32 | 27 | 3 | 12 |
| 35926 | 35926 | 0 | 29.50% | F1 | 31 | 26 | 2 | 13 |
| 37603.5 | 37603.5 | 0 | 29.40% | F1 | 34 | 29 | 0 | 16 |
| 36682.7 | 36683 | 8 | 29.10% | S | 31 | 26 | 3 | 15 |
| 37484.4 | 37484.6 | 5 | 28.40% | S | 32 | 27 | 2 | 17 |
| 36900.9 | 36901 | 3 | 28.20% | S | 32 | 27 | 0 | 16 |
| 35882 | 35882 | 0 | 28.10% | S | 32 | 27 | 1 | 12 |
| 37748.7 | 37749 | 8 | 28.00% | F1 | 32 | 27 | 4 | 17 |
| 38770.6 | 38771.5 | 23 | 27.90% | F1 | 34 | 29 | 4 | 18 |
| 35632.7 | 35633 | 8 | 27.80% | F1 | 29 | 24 | 3 | 14 |
| 36828.9 | 36829 | 3 | 27.60% | S | 31 | 26 | 4 | 15 |
| 37820.7 | 37821 | 8 | 27.50% | F1 | 33 | 28 | 0 | 18 |
| 35662.8 | 35663 | 6 | 27.10% | S | 31 | 26 | 2 | 12 |
| 36247.3 | 36247.5 | 6 | 27.00% | S | 33 | 28 | 1 | 12 |
| 38333.2 | 38333.5 | 8 | 27.00% | F1 | 34 | 29 | 3 | 17 |
| 38141 | 38141.5 | 13 | 26.80% | S | 33 | 28 | 2 | 18 |
| 34978.2 | 34978.5 | 9 | 26.00% | F1 | 30 | 25 | 2 | 11 |
| 36173.3 | 36173.5 | 6 | 25.70% | S | 32 | 27 | 1 | 13 |
| 36100.2 | 36100.5 | 8 | 25.60% | S | 31 | 26 | 3 | 13 |
| 34759 | 34759.1 | 3 | 25.20% | F1 | 29 | 24 | 3 | 11 |
| 36319.4 | 36319.9 | 14 | 24.30% | S | 32 | 27 | 2 | 13 |
| 34789.1 | 34789 | -3 | 24.10% | S | 31 | 26 | 2 | 9 |
| 38434.3 | 38434.6 | 8 | 24.00% | S | 35 | 30 | 1 | 17 |
| 34834 | 34834 | 0 | 23.20% | F1 | 32 | 27 | 0 | 9 |
| 35154.4 | 35154 | -11 | 22.90% | S | 32 | 27 | 2 | 9 |
| 34104.4 | 34104 | -12 | 21.50% | F1 | 30 | 25 | 2 | 8 |
| 35518.7 | 35518.6 | -3 | 20.90% | S | 33 | 28 | 0 | 10 |
| 35340.5 | 35340.4 | -3 | 20.70% | F1 | 29 | 24 | 1 | 14 |
| 38479.3 | 38479 | -8 | 20.30% | F1 | 34 | 29 | 4 | 17 |
| 34614.9 | 34614.9 | 0 | 20.00% | F1 | 31 | 26 | 1 | 9 |
| 35125.3 | 35125.5 | 6 | 19.10% | F1 | 32 | 27 | 0 | 10 |
| 33959.3 | 33959.5 | 6 | 17.70% | F1 | 30 | 25 | 3 | 7 |
| 36362.4 | 36362.7 | 8 | 17.50% | F1 | 31 | 26 | 1 | 15 |
| 34323.6 | 34324.1 | 15 | 17.30% | F1 | 31 | 26 | 1 | 8 |
| 34029.3 | 34028.9 | -12 | 17.20% | F1 | 29 | 24 | 0 | 10 |
| 35781.9 | 35781.5 | -11 | 17.10% | F1 | 33 | 28 | 0 | 11 |
| 33258.7 | 33259 | 9 | 16.90% | F2 | 26 | 21 | 4 | 9 |
| 33841.2 | 33841.5 | 9 | 16.80% | S | 30 | 25 | 2 | 7 |
| 38580.4 | 38580 | -10 | 15.50% | S | 35 | 30 | 2 | 17 |
| 35416.6 | 35416.4 | -6 | 15.10% | F1 | 32 | 27 | 0 | 11 |
| 35300.5 | 35300.5 | 0 | 14.80% | S | 32 | 27 | 3 | 9 |
| 35664.8 | 35665.1 | 8 | 14.00% | S | 33 | 28 | 1 | 10 |
| 35489.6 | 35489.5 | -3 | 13.20% | F1 | 31 | 26 | 3 | 11 |
| 33156.6 | 33156.4 | -6 | 12.60% | F1 | 29 | 24 | 2 | 6 |
| 33813.2 | 33813.5 | 9 | 12.60% | F1 | 30 | 25 | 2 | 7 |
| 34249.5 | 34250 | 15 | 12.50% | F1 | 30 | 25 | 1 | 9 |
| 35081.3 | 35081.1 | -6 | 12.20% | S | 31 | 26 | 4 | 9 |
| 34206.5 | 34206.5 | 0 | 12.10% | F2 | 27 | 22 | 4 | 11 |
| 34027.3 | 34026.7 | -18 | 11.50% | F1 | 27 | 22 | 1 | 12 |
| 37994.9 | 37994.2 | -18 | 11.50% | S | 33 | 28 | 1 | 18 |
| 34497.8 | 34497.6 | -6 | 11.50% | S | 31 | 26 | 2 | 8 |
| 33549.9 | 33549.5 | -12 | 11.40% | F2 | 26 | 21 | 4 | 10 |
| 33885.2 | 33885 | -6 | 10.90% | F1 | 29 | 24 | 3 | 8 |
| 34541.8 | 34542.1 | 9 | 10.60% | F1 | 30 | 25 | 3 | 9 |
| 34278.6 | 34279.1 | 15 | 10.60% | S | 30 | 25 | 3 | 8 |
| 34643.9 | 34644 | 3 | 10.50% | S | 31 | 26 | 3 | 8 |
| 34423.7 | 34424 | 9 | 10.40% | S | 30 | 25 | 2 | 9 |
| 34687.9 | 34687.6 | -9 | 10.30% | F1 | 30 | 25 | 4 | 9 |
| 35226.5 | 35226.6 | 3 | 10.00% | S | 31 | 26 | 3 | 10 |
| 32790.2 | 32790.1 | -3 | 9.90% | F1 | 28 | 23 | 0 | 7 |
| 38214.1 | 38214 | -3 | 9.80% | S | 34 | 29 | 0 | 18 |
| 32864.3 | 32864.5 | 6 | 9.70% | F1 | 29 | 24 | 0 | 6 |
| 38651.5 | 38651 | -13 | 9.70% | S | 34 | 29 | 1 | 19 |
| 33184.6 | 33185 | 12 | 9.60% | S | 29 | 24 | 2 | 6 |
| 35009.2 | 35009.4 | 6 | 9.50% | F2 | 30 | 25 | 0 | 12 |
| 37413.4 | 37413.6 | 5 | 9.40% | S | 33 | 28 | 3 | 15 |
| 34718 | 34718 | 0 | 9.30% | F2 | 30 | 25 | 0 | 11 |
| 38725.5 | 38724.6 | -23 | 9.30% | S | 35 | 30 | 1 | 18 |
| 34319.6 | 34319.2 | -12 | 8.70% | F1 | 27 | 22 | 3 | 12 |
| 33331.7 | 33332 | 9 | 8.60% | F2 | 27 | 22 | 2 | 9 |
| 34935.1 | 34935.1 | 0 | 8.40% | F2 | 29 | 24 | 0 | 13 |
| 33476.8 | 33476.5 | -9 | 8.40% | F2 | 27 | 22 | 1 | 10 |
| 33696 | 33696.5 | 15 | 8.00% | F2 | 28 | 23 | 0 | 10 |
| 37996.9 | 37997 | 3 | 8.00% | S | 35 | 30 | 0 | 16 |
| 34061.4 | 34061.5 | 3 | 7.90% | F2 | 29 | 24 | 0 | 10 |
| 33914.2 | 33914.4 | 6 | 7.80% | F2 | 27 | 22 | 2 | 11 |
| 34860.1 | 34860.5 | 11 | 7.80% | S | 30 | 25 | 1 | 11 |
| 33373.7 | 33374 | 9 | 7.70% | F1 | 28 | 23 | 2 | 8 |
| 34570.8 | 34570.6 | -6 | 7.60% | F2 | 28 | 23 | 2 | 12 |
| 33520.9 | 33521 | 3 | 7.60% | F1 | 30 | 25 | 0 | 7 |
| 35008.2 | 35008.5 | 9 | 7.40% | S | 32 | 27 | 1 | 9 |
| 34133.5 | 34134 | 15 | 7.00% | S | 30 | 25 | 4 | 7 |
| 34466.7 | 34466.5 | -6 | 6.80% | F1 | 29 | 24 | 1 | 11 |
| 33010.4 | 33011 | 18 | 6.80% | F1 | 29 | 24 | 1 | 6 |
| 34934.2 | 34934.5 | 9 | 6.70% | S | 31 | 26 | 1 | 10 |
| 33769.1 | 33769.5 | 12 | 6.60% | F2 | 27 | 22 | 3 | 10 |
| 34977.1 | 34977 | -3 | 6.60% | F1 | 30 | 25 | 0 | 12 |
| 34424.7 | 34424.5 | -6 | 6.30% | S | 30 | 25 | 4 | 8 |
| 33445.8 | 33445.5 | -9 | 6.30% | F1 | 27 | 22 | 3 | 9 |
| 33623 | 33623 | 0 | 6.30% | F2 | 27 | 22 | 2 | 10 |
| 34425.7 | 34426 | 9 | 6.00% | F2 | 28 | 23 | 3 | 11 |
| 33739.1 | 33739.5 | 12 | 5.90% | F1 | 29 | 24 | 2 | 8 |
| 37923.8 | 37923.7 | -3 | 5.90% | S | 34 | 29 | 2 | 16 |
| 33987.3 | 33987.1 | -6 | 5.80% | F2 | 28 | 23 | 0 | 11 |
| 34132.5 | 34132.5 | 0 | 5.80% | S | 30 | 25 | 2 | 8 |
| 34135.4 | 34135 | -12 | 5.80% | F2 | 30 | 25 | 0 | 9 |
| 34352.6 | 34352.5 | -3 | 5.60% | F2 | 29 | 24 | 0 | 11 |
| 36946.9 | 36947.4 | 14 | 5.60% | F1 | 33 | 28 | 0 | 15 |
| 37530.4 | 37530.5 | 3 | 5.60% | F1 | 33 | 28 | 2 | 16 |
| 33404.8 | 33404.5 | -9 | 5.60% | F2 | 28 | 23 | 0 | 9 |
| 33666 | 33666.5 | 15 | 5.20% | F1 | 28 | 23 | 4 | 8 |
| 37850.8 | 37851.2 | 11 | 4.70% | S | 33 | 28 | 4 | 16 |
| 33302.7 | 33302.6 | -3 | 4.60% | F1 | 29 | 24 | 3 | 6 |
| 34177.5 | 34177.6 | 3 | 4.50% | F1 | 31 | 26 | 0 | 8 |
| 38843.6 | 38844 | 10 | 4.40% | F1 | 35 | 30 | 2 | 18 |
| 34394.6 | 34394.5 | -3 | 4.40% | F1 | 30 | 25 | 0 | 10 |
| 37966.8 | 37966.5 | -8 | 2.20% | F1 | 33 | 28 | 1 | 18 |

^a^Relative intensity based on the intensities of the intact masses after deconvolution.

^b^total number of hexoses (Hex), N-acetylhexosamine (HexNAc), fucose (Fuc) and sialic acids (SA)

Table S6. Putatively assigned AGP glycoforms by nano-CZE-MS cut 1 (ordered by decreasing relative intensity).

| Theoretical Mass [Da] | Observed Mass [Da] | Deviation [ppm] | Rel. Int^a^ | Variant | Glycosylation^b^ | | | |
| --- | --- | --- | --- | --- | --- | --- | --- | --- |
|  |  |  |  |  | Hex | HexNac | Fuc | SA |
| 37676.6 | 37676.5 | -3 | 100.00% | F1 | 33 | 28 | 3 | 16 |
| 36217.3 | 36217.4 | 3 | 99.20% | F1 | 31 | 26 | 2 | 14 |
| 35955.1 | 35955.1 | 0 | 90.60% | S | 31 | 26 | 4 | 12 |
| 36145.2 | 36144.9 | -8 | 90.50% | F1 | 32 | 27 | 1 | 13 |
| 37558.5 | 37558.9 | 11 | 87.90% | S | 33 | 28 | 2 | 16 |
| 35560.7 | 35561 | 8 | 86.00% | F1 | 30 | 25 | 2 | 13 |
| 36072.2 | 36072.5 | 8 | 86.00% | F1 | 31 | 26 | 3 | 13 |
| 36436.5 | 36437 | 14 | 83.70% | F1 | 32 | 27 | 1 | 14 |
| 35998.1 | 35998.5 | 11 | 81.90% | F1 | 30 | 25 | 3 | 14 |
| 36144.2 | 36144.5 | 8 | 77.40% | F1 | 30 | 25 | 4 | 14 |
| 36245.3 | 36245.5 | 6 | 76.60% | S | 31 | 26 | 2 | 14 |
| 36536.6 | 36537 | 11 | 75.40% | S | 31 | 26 | 2 | 15 |
| 36364.4 | 36364.5 | 3 | 75.40% | F1 | 33 | 28 | 0 | 13 |
| 35880 | 35880 | 0 | 73.90% | S | 30 | 25 | 2 | 14 |
| 36071.1 | 36071.5 | 11 | 73.40% | F1 | 31 | 26 | 1 | 14 |
| 36610.7 | 36611 | 8 | 72.20% | S | 32 | 27 | 2 | 14 |
| 37384.3 | 37384 | -8 | 70.10% | F1 | 33 | 28 | 1 | 16 |
| 35195.4 | 35195.5 | 3 | 70.10% | F1 | 29 | 24 | 2 | 13 |
| 35853 | 35853.5 | 14 | 64.20% | F1 | 30 | 25 | 4 | 13 |
| 35269.4 | 35269.1 | -9 | 63.80% | F1 | 30 | 25 | 2 | 12 |
| 36435.5 | 36435.5 | 0 | 63.00% | F1 | 30 | 25 | 4 | 15 |
| 34904.1 | 34904.4 | 9 | 59.00% | F1 | 29 | 24 | 2 | 12 |
| 36799.8 | 36799 | -22 | 58.80% | F1 | 31 | 26 | 2 | 16 |
| 36246.4 | 36246.5 | 3 | 57.00% | S | 31 | 26 | 4 | 13 |
| 36465.5 | 36466 | 14 | 55.90% | S | 32 | 27 | 3 | 13 |
| 35486.6 | 35487 | 11 | 55.40% | F1 | 29 | 24 | 2 | 14 |
| 38552.4 | 38552 | -10 | 54.70% | F1 | 35 | 30 | 2 | 17 |
| 37849.8 | 37850 | 5 | 54.10% | S | 33 | 28 | 2 | 17 |
| 36947.9 | 36947.5 | -11 | 53.60% | F1 | 33 | 28 | 2 | 14 |
| 38260.1 | 38260.1 | 0 | 52.60% | F1 | 35 | 30 | 0 | 17 |
| 37704.6 | 37705 | 11 | 51.70% | S | 33 | 28 | 3 | 16 |
| 35589.8 | 35589.9 | 3 | 51.20% | S | 30 | 25 | 4 | 12 |
| 36537.6 | 36537.5 | -3 | 50.10% | S | 31 | 26 | 4 | 14 |
| 37967.8 | 37967.5 | -8 | 49.90% | F1 | 33 | 28 | 3 | 17 |
| 34686.9 | 34686.5 | -12 | 49.80% | F1 | 30 | 25 | 2 | 10 |
| 37457.4 | 37457.5 | 3 | 49.50% | F1 | 32 | 27 | 4 | 16 |
| 36509.6 | 36510 | 11 | 49.30% | F1 | 31 | 26 | 4 | 14 |
| 36538.6 | 36539 | 11 | 47.20% | S | 33 | 28 | 1 | 13 |
| 36391.5 | 36392 | 14 | 47.10% | S | 31 | 26 | 3 | 14 |
| 35445.6 | 35446 | 11 | 44.00% | S | 32 | 27 | 2 | 10 |
| 37456.4 | 37456.5 | 3 | 43.50% | F1 | 32 | 27 | 2 | 17 |
| 35881 | 35881 | 0 | 42.60% | S | 30 | 25 | 4 | 13 |
| 37048.1 | 37048.1 | 0 | 41.60% | S | 32 | 27 | 3 | 15 |
| 37339.3 | 37339.5 | 5 | 41.40% | S | 32 | 27 | 3 | 16 |
| 37238.2 | 37237.5 | -19 | 41.20% | F1 | 33 | 28 | 0 | 16 |
| 36684.7 | 36684.5 | -5 | 41.00% | S | 33 | 28 | 2 | 13 |
| 36975 | 36975.5 | 14 | 40.10% | S | 33 | 28 | 0 | 15 |
| 35735.9 | 35735.5 | -11 | 40.10% | S | 32 | 27 | 0 | 12 |
| 36801.8 | 36801.5 | -8 | 40.10% | F1 | 33 | 28 | 1 | 14 |
| 38187 | 38186.6 | -10 | 40.00% | F1 | 34 | 29 | 2 | 17 |
| 36027.1 | 36027 | -3 | 39.60% | S | 32 | 27 | 0 | 13 |
| 36581.6 | 36581 | -16 | 39.50% | F1 | 32 | 27 | 0 | 15 |
| 35954.1 | 35954 | -3 | 39.30% | S | 31 | 26 | 2 | 13 |
| 37995.9 | 37996 | 3 | 39.30% | S | 33 | 28 | 3 | 17 |
| 36873.9 | 36873.9 | 0 | 38.70% | F1 | 32 | 27 | 2 | 15 |
| 36756.8 | 36756.5 | -8 | 38.50% | S | 32 | 27 | 3 | 14 |
| 35780.9 | 35780.5 | -11 | 37.90% | F1 | 31 | 26 | 3 | 12 |
| 36727.7 | 36727.9 | 5 | 37.10% | F1 | 32 | 27 | 1 | 15 |
| 37894.8 | 37895 | 5 | 37.10% | F1 | 34 | 29 | 0 | 17 |
| 35883 | 35883 | 0 | 36.80% | S | 32 | 27 | 3 | 11 |
| 38989.8 | 38990.5 | 18 | 36.50% | F1 | 35 | 30 | 3 | 18 |
| 38289.2 | 38289 | -5 | 36.40% | S | 35 | 30 | 2 | 16 |
| 36026.1 | 36026.1 | 0 | 36.30% | S | 30 | 25 | 3 | 14 |
| 35268.4 | 35268.6 | 6 | 36.30% | F1 | 30 | 25 | 0 | 13 |
| 37194.2 | 37194.5 | 8 | 35.50% | S | 32 | 27 | 4 | 15 |
| 35955.1 | 35955.1 | 0 | 100.10% | S | 31 | 26 | 4 | 12 |
| 36249.3 | 36249.4 | 3 | 99.10% | F2 | 31 | 26 | 2 | 14 |
| 35592.7 | 35592.6 | -3 | 95.10% | F2 | 30 | 25 | 2 | 13 |
| 36320.4 | 36320.6 | 6 | 92.90% | S | 32 | 27 | 4 | 12 |
| 35884 | 35884.1 | 3 | 88.50% | F2 | 30 | 25 | 2 | 14 |
| 35302.5 | 35302.1 | -11 | 84.30% | F2 | 30 | 25 | 4 | 11 |
| 36541.6 | 36541.5 | -3 | 84.30% | F2 | 31 | 26 | 4 | 14 |
| 35958.1 | 35958.5 | 11 | 83.60% | F2 | 31 | 26 | 2 | 13 |
| 35663.8 | 35664 | 6 | 75.70% | S | 31 | 26 | 4 | 11 |
| 35737.9 | 35738 | 3 | 75.40% | S | 32 | 27 | 4 | 10 |
| 36540.6 | 36540.5 | -3 | 74.60% | F2 | 31 | 26 | 2 | 15 |
| 36175.3 | 36175.5 | 6 | 71.10% | S | 34 | 29 | 0 | 11 |
| 35666.8 | 35667 | 6 | 71.10% | F2 | 31 | 26 | 2 | 12 |
| 36903.9 | 36904 | 3 | 65.20% | S | 34 | 29 | 1 | 13 |
| 34644.9 | 34644.5 | -12 | 65.00% | F2 | 29 | 24 | 2 | 11 |
| 36031.1 | 36031 | -3 | 64.70% | F2 | 32 | 27 | 0 | 13 |
| 34936.2 | 34936 | -6 | 63.10% | F2 | 29 | 24 | 2 | 12 |
| 36612.7 | 36613 | 8 | 61.60% | S | 34 | 29 | 1 | 12 |
| 35011.3 | 35011 | -9 | 61.00% | F2 | 30 | 25 | 4 | 10 |
| 34353.7 | 34354 | 9 | 59.80% | F2 | 29 | 24 | 2 | 10 |
| 35375.6 | 35375.5 | -3 | 58.20% | F2 | 31 | 26 | 2 | 11 |
| 36395.5 | 36396 | 14 | 57.00% | F2 | 31 | 26 | 3 | 14 |
| 36394.4 | 36394 | -11 | 55.70% | F2 | 31 | 26 | 1 | 15 |
| 33916.3 | 33916 | -9 | 55.30% | F2 | 29 | 24 | 1 | 9 |
| 36685.8 | 36686 | 5 | 55.20% | S | 33 | 28 | 4 | 12 |
| 35446.7 | 35447.1 | 11 | 55.00% | S | 32 | 27 | 4 | 9 |
| 36104.2 | 36104.5 | 8 | 53.80% | F2 | 31 | 26 | 3 | 13 |
| 36323.4 | 36323.5 | 3 | 53.50% | F2 | 32 | 27 | 2 | 13 |
| 36979 | 36979.5 | 14 | 51.80% | F2 | 33 | 28 | 0 | 15 |
| 35738.9 | 35738.5 | -11 | 51.70% | F2 | 30 | 25 | 3 | 13 |
| 36322.4 | 36322.5 | 3 | 50.90% | F2 | 32 | 27 | 0 | 14 |
| 36977 | 36977 | 0 | 49.80% | S | 33 | 28 | 4 | 13 |
| 35445.6 | 35446 | 11 | 48.60% | S | 32 | 27 | 2 | 10 |
| 34790 | 34790 | 0 | 47.80% | F2 | 29 | 24 | 1 | 12 |
| 34863.1 | 34863 | -3 | 47.60% | F2 | 28 | 23 | 4 | 12 |
| 37199.2 | 37199.1 | -3 | 46.40% | F2 | 34 | 29 | 1 | 14 |
| 36831.9 | 36832 | 3 | 46.30% | S | 35 | 30 | 0 | 12 |
| 36905.9 | 36906 | 3 | 44.90% | F2 | 32 | 27 | 2 | 15 |
| 36466.5 | 36467 | 14 | 44.60% | S | 34 | 29 | 0 | 12 |
| 34719 | 34719.4 | 12 | 44.20% | F2 | 30 | 25 | 2 | 10 |
| 36614.7 | 36615 | 8 | 44.10% | F2 | 32 | 27 | 2 | 14 |
| 35883 | 35883 | 0 | 40.60% | S | 32 | 27 | 3 | 11 |
| 36321.4 | 36321.5 | 3 | 40.40% | S | 34 | 29 | 1 | 11 |
| 36613.6 | 36613.5 | -3 | 40.30% | F2 | 32 | 27 | 0 | 15 |
| 33988.3 | 33988.4 | 3 | 38.40% | F2 | 28 | 23 | 2 | 10 |
| 34790.1 | 34790.5 | 11 | 37.90% | S | 31 | 26 | 4 | 8 |
| 35084.3 | 35084.5 | 6 | 37.30% | F2 | 31 | 26 | 2 | 10 |
| 35811 | 35810.5 | -14 | 36.90% | S | 33 | 28 | 2 | 10 |
| 36757.8 | 36758 | 5 | 35.60% | S | 34 | 29 | 0 | 13 |
| 35372.5 | 35372.1 | -11 | 35.50% | F2 | 29 | 24 | 1 | 14 |
| 35372.6 | 35373 | 11 | 35.30% | S | 31 | 26 | 4 | 10 |
| 37271.3 | 37271.5 | 5 | 35.20% | F2 | 33 | 28 | 2 | 15 |
| 37490.4 | 37490.6 | 5 | 34.20% | F2 | 34 | 29 | 1 | 15 |
| 36102.2 | 36102 | -6 | 33.50% | S | 33 | 28 | 2 | 11 |
| 34498.8 | 34499.1 | 9 | 33.00% | F2 | 29 | 24 | 1 | 11 |
| 37126.1 | 37126.1 | 0 | 32.20% | F2 | 33 | 28 | 3 | 14 |
| 37197.2 | 37197.5 | 8 | 30.70% | F2 | 32 | 27 | 2 | 16 |
| 33550.9 | 33551 | 3 | 29.90% | F2 | 28 | 23 | 1 | 9 |
| 36978 | 36977.9 | -3 | 28.40% | S | 35 | 30 | 1 | 12 |
| 37051.1 | 37051.5 | 11 | 28.20% | S | 34 | 29 | 4 | 12 |
| 34427.7 | 34427.5 | -6 | 28.00% | F2 | 30 | 25 | 2 | 9 |
| 34717 | 34717.5 | 14 | 26.60% | F2 | 28 | 23 | 3 | 12 |
| 35154.4 | 35154 | -11 | 25.30% | S | 32 | 27 | 2 | 9 |
| 37562.5 | 37563 | 13 | 23.90% | F2 | 33 | 28 | 2 | 16 |
| 35227.4 | 35227.5 | 3 | 23.30% | F2 | 29 | 24 | 2 | 13 |
| 34062.4 | 34062.6 | 6 | 23.20% | F2 | 29 | 24 | 2 | 9 |
| 33770.1 | 33770 | -3 | 23.20% | F2 | 29 | 24 | 0 | 9 |
| 35518.7 | 35518.6 | -3 | 23.10% | S | 33 | 28 | 0 | 10 |
| 36248.4 | 36248.5 | 3 | 22.60% | S | 33 | 28 | 3 | 11 |
| 34500.8 | 34500.9 | 3 | 19.10% | F2 | 31 | 26 | 0 | 9 |
| 33258.7 | 33259 | 9 | 18.70% | F2 | 26 | 21 | 4 | 9 |
| 37051 | 37050.6 | -11 | 18.70% | F2 | 32 | 27 | 1 | 16 |
| 37417.4 | 37417.1 | -8 | 18.60% | F2 | 33 | 28 | 3 | 15 |
| 37124.1 | 37124 | -3 | 18.60% | F2 | 31 | 26 | 4 | 16 |
| 37343.3 | 37343.5 | 5 | 17.60% | F2 | 32 | 27 | 3 | 16 |
| 33697.1 | 33697 | -3 | 16.80% | F2 | 28 | 23 | 2 | 9 |
| 35300.5 | 35300.5 | 0 | 16.30% | S | 32 | 27 | 3 | 9 |
| 35664.8 | 35665.1 | 8 | 15.50% | S | 33 | 28 | 1 | 10 |
| 37416.4 | 37416.5 | 3 | 15.30% | S | 35 | 30 | 4 | 12 |
| 35081.3 | 35081.1 | -6 | 13.50% | S | 31 | 26 | 4 | 9 |
| 34205.5 | 34205.6 | 3 | 13.40% | S | 31 | 26 | 0 | 8 |
| 34278.6 | 34279.1 | 15 | 11.70% | S | 30 | 25 | 3 | 8 |
| 35009.2 | 35009.4 | 6 | 10.50% | F2 | 30 | 25 | 0 | 12 |
| 37196.1 | 37196 | -3 | 10.00% | F2 | 32 | 27 | 0 | 17 |
| 37270.2 | 37270 | -5 | 9.70% | F2 | 33 | 28 | 0 | 16 |
| 33331.7 | 33332 | 9 | 9.40% | F2 | 27 | 22 | 2 | 9 |
| 36029.1 | 36029 | -3 | 9.20% | F2 | 30 | 25 | 1 | 15 |
| 33696 | 33696.5 | 15 | 8.90% | F2 | 28 | 23 | 0 | 10 |
| 34061.4 | 34061.5 | 3 | 8.70% | F2 | 29 | 24 | 0 | 10 |
| 34570.8 | 34570.6 | -6 | 8.40% | F2 | 28 | 23 | 2 | 12 |
| 34133.5 | 34134 | 15 | 7.70% | S | 30 | 25 | 4 | 7 |
| 33623 | 33623 | 0 | 6.90% | F2 | 27 | 22 | 2 | 10 |
| 34425.7 | 34426 | 9 | 6.60% | F2 | 28 | 23 | 3 | 11 |
| 33698.1 | 33698.5 | 12 | 6.60% | F2 | 28 | 23 | 4 | 8 |
| 33842.2 | 33842.5 | 9 | 6.20% | F2 | 28 | 23 | 1 | 10 |
| 34352.6 | 34352.5 | -3 | 6.20% | F2 | 29 | 24 | 0 | 11 |
| 37635.6 | 37635.5 | -3 | 6.20% | F2 | 34 | 29 | 0 | 16 |
| 33405.8 | 33405.4 | -12 | 4.80% | F2 | 28 | 23 | 2 | 8 |
| 37342.3 | 37342 | -8 | 4.80% | F2 | 32 | 27 | 1 | 17 |
| 33332.8 | 33333 | 6 | 2.90% | F2 | 27 | 22 | 4 | 8 |
| 34977.1 | 34977 | -3 | 6.60% | F1 | 30 | 25 | 0 | 12 |
| 34424.7 | 34424.5 | -6 | 6.30% | S | 30 | 25 | 4 | 8 |
| 33445.8 | 33445.5 | -9 | 6.30% | F1 | 27 | 22 | 3 | 9 |
| 33623 | 33623 | 0 | 6.30% | F2 | 27 | 22 | 2 | 10 |
| 34425.7 | 34426 | 9 | 6.00% | F2 | 28 | 23 | 3 | 11 |
| 33739.1 | 33739.5 | 12 | 5.90% | F1 | 29 | 24 | 2 | 8 |
| 37923.8 | 37923.7 | -3 | 5.90% | S | 34 | 29 | 2 | 16 |
| 33987.3 | 33987.1 | -6 | 5.80% | F2 | 28 | 23 | 0 | 11 |
| 34132.5 | 34132.5 | 0 | 5.80% | S | 30 | 25 | 2 | 8 |
| 34135.4 | 34135 | -12 | 5.80% | F2 | 30 | 25 | 0 | 9 |
| 34352.6 | 34352.5 | -3 | 5.60% | F2 | 29 | 24 | 0 | 11 |
| 36946.9 | 36947.4 | 14 | 5.60% | F1 | 33 | 28 | 0 | 15 |
| 37530.4 | 37530.5 | 3 | 5.60% | F1 | 33 | 28 | 2 | 16 |
| 33404.8 | 33404.5 | -9 | 5.60% | F2 | 28 | 23 | 0 | 9 |
| 33666 | 33666.5 | 15 | 5.20% | F1 | 28 | 23 | 4 | 8 |
| 37850.8 | 37851.2 | 11 | 4.70% | S | 33 | 28 | 4 | 16 |
| 33302.7 | 33302.6 | -3 | 4.60% | F1 | 29 | 24 | 3 | 6 |
| 34177.5 | 34177.6 | 3 | 4.50% | F1 | 31 | 26 | 0 | 8 |
| 38843.6 | 38844 | 10 | 4.40% | F1 | 35 | 30 | 2 | 18 |
| 34394.6 | 34394.5 | -3 | 4.40% | F1 | 30 | 25 | 0 | 10 |
| 37966.8 | 37966.5 | -8 | 2.20% | F1 | 33 | 28 | 1 | 18 |

^a^Relative intensity based on the intensities of the intact masses after deconvolution.

^b^total number of hexoses (Hex), N-acetylhexosamine (HexNAc), fucose (Fuc) and sialic acids (SA)

Table S7. Putatively assigned AGP glycoforms by nano-CZE-MS cut 2 (ordered by decreasing relative intensity).

| Theoretical Mass [Da] | Observed Mass [Da] | Deviation [ppm] | Rel. Int^a^ | Variant | Glycosylation^b^ | | | |
| --- | --- | --- | --- | --- | --- | --- | --- | --- |
|  |  |  |  |  | Hex | HexNac | Fuc | SA |
| 37676.6 | 37676.5 | -3 | 100.00% | F1 | 33 | 28 | 3 | 16 |
| 35635.8 | 35635.5 | -8 | 100.00% | F1 | 31 | 26 | 4 | 11 |
| 36217.3 | 36217.4 | 3 | 99.20% | F1 | 31 | 26 | 2 | 14 |
| 35490.6 | 35490.5 | -3 | 91.60% | F1 | 33 | 28 | 0 | 10 |
| 35955.1 | 35955.1 | 0 | 90.60% | S | 31 | 26 | 4 | 12 |
| 36145.2 | 36144.9 | -8 | 90.50% | F1 | 32 | 27 | 1 | 13 |
| 37558.5 | 37558.9 | 11 | 87.90% | S | 33 | 28 | 2 | 16 |
| 35560.7 | 35561 | 8 | 86.00% | F1 | 30 | 25 | 2 | 13 |
| 36072.2 | 36072.5 | 8 | 86.00% | F1 | 31 | 26 | 3 | 13 |
| 35927 | 35926.9 | -3 | 85.80% | F1 | 31 | 26 | 4 | 12 |
| 36436.5 | 36437 | 14 | 83.70% | F1 | 32 | 27 | 1 | 14 |
| 35998.1 | 35998.5 | 11 | 81.90% | F1 | 30 | 25 | 3 | 14 |
| 35198.4 | 35198.5 | 3 | 77.00% | F1 | 31 | 26 | 3 | 10 |
| 36028.2 | 36028.5 | 8 | 75.20% | S | 32 | 27 | 2 | 12 |
| 36291.3 | 36291 | -8 | 72.90% | F1 | 32 | 27 | 2 | 13 |
| 36145.2 | 36144.9 | -8 | 71.90% | F1 | 32 | 27 | 1 | 13 |
| 36249.3 | 36249.4 | 3 | 71.30% | F2 | 31 | 26 | 2 | 14 |
| 35560.7 | 35561 | 8 | 68.30% | F1 | 30 | 25 | 2 | 13 |
| 36655.7 | 36655.5 | -5 | 67.80% | F1 | 33 | 28 | 0 | 14 |
| 36320.4 | 36320.6 | 6 | 66.80% | S | 32 | 27 | 4 | 12 |
| 35153.4 | 35153.9 | 14 | 65.80% | S | 32 | 27 | 0 | 10 |
| 36366.5 | 36367 | 14 | 65.50% | F1 | 33 | 28 | 4 | 11 |
| 35053.3 | 35053 | -9 | 60.90% | F1 | 31 | 26 | 4 | 9 |
| 36536.6 | 36537 | 11 | 59.90% | S | 31 | 26 | 2 | 15 |
| 34980.2 | 34981.1 | 26 | 58.90% | F1 | 32 | 27 | 1 | 9 |
| 35880 | 35880 | 0 | 58.70% | S | 30 | 25 | 2 | 14 |
| 36610.7 | 36611 | 8 | 57.30% | S | 32 | 27 | 2 | 14 |
| 36001.1 | 36001.5 | 11 | 55.60% | F1 | 32 | 27 | 4 | 11 |
| 34615.9 | 34616 | 3 | 55.50% | F1 | 31 | 26 | 3 | 8 |
| 35663.8 | 35664 | 6 | 54.50% | S | 31 | 26 | 4 | 11 |
| 36585.6 | 36585.5 | -3 | 53.50% | F1 | 34 | 29 | 3 | 11 |
| 35956.1 | 35956 | -3 | 51.40% | S | 33 | 28 | 1 | 11 |
| 36143.2 | 36142.5 | -19 | 51.40% | F1 | 30 | 25 | 2 | 15 |
| 35269.4 | 35269.1 | -9 | 50.70% | F1 | 30 | 25 | 2 | 12 |
| 35371.6 | 35372 | 11 | 49.40% | S | 31 | 26 | 2 | 11 |
| 35270.5 | 35270.9 | 11 | 48.00% | F1 | 30 | 25 | 4 | 11 |
| 34906.1 | 34906.6 | 14 | 46.00% | F1 | 31 | 26 | 1 | 10 |
| 36246.4 | 36246.5 | 3 | 45.30% | S | 31 | 26 | 4 | 13 |
| 37267.2 | 37267 | -5 | 43.10% | S | 33 | 28 | 2 | 15 |
| 36073.1 | 36073.5 | 11 | 41.70% | F1 | 33 | 28 | 0 | 12 |
| 35299.5 | 35299.5 | 0 | 39.30% | S | 32 | 27 | 1 | 10 |
| 35444.6 | 35445 | 11 | 38.40% | S | 32 | 27 | 0 | 11 |
| 36538.6 | 36539 | 11 | 37.50% | S | 33 | 28 | 1 | 13 |
| 36391.5 | 36392 | 14 | 37.40% | S | 31 | 26 | 3 | 14 |
| 35854 | 35854.4 | 11 | 35.20% | F1 | 32 | 27 | 1 | 12 |
| 36219.3 | 36219.1 | -6 | 34.80% | F1 | 33 | 28 | 1 | 12 |
| 36101.2 | 36101.1 | -3 | 34.60% | S | 33 | 28 | 0 | 12 |
| 34496.8 | 34497.1 | 9 | 34.20% | S | 31 | 26 | 0 | 9 |
| 36657.7 | 36658.5 | 22 | 33.60% | F1 | 33 | 28 | 4 | 12 |
| 36831.9 | 36832 | 3 | 33.30% | S | 35 | 30 | 0 | 12 |
| 37049 | 37049.5 | 13 | 32.70% | S | 34 | 29 | 0 | 14 |
| 35123.3 | 35123.6 | 9 | 32.50% | F1 | 30 | 25 | 1 | 12 |
| 36147.2 | 36147 | -6 | 32.40% | F1 | 34 | 29 | 0 | 11 |
| 36466.5 | 36467 | 14 | 32.10% | S | 34 | 29 | 0 | 12 |
| 36975 | 36975.5 | 14 | 31.90% | S | 33 | 28 | 0 | 15 |
| 36027.1 | 36027 | -3 | 31.40% | S | 32 | 27 | 0 | 13 |
| 35999.1 | 35999 | -3 | 30.80% | F1 | 32 | 27 | 0 | 13 |
| 36756.8 | 36756.5 | -8 | 30.60% | S | 32 | 27 | 3 | 14 |
| 36583.6 | 36584 | 11 | 30.40% | F1 | 32 | 27 | 4 | 13 |
| 35122.3 | 35121.5 | -23 | 30.00% | F1 | 28 | 23 | 4 | 13 |
| 36803.8 | 36804.5 | 19 | 29.50% | F1 | 35 | 30 | 0 | 12 |
| 35883 | 35883 | 0 | 29.20% | S | 32 | 27 | 3 | 11 |
| 34422.7 | 34423 | 9 | 29.10% | S | 30 | 25 | 0 | 10 |
| 36950 | 36950.5 | 14 | 26.60% | F1 | 35 | 30 | 1 | 12 |
| 35811 | 35810.5 | -14 | 26.60% | S | 33 | 28 | 2 | 10 |
| 35051.2 | 35051 | -6 | 26.40% | F1 | 31 | 26 | 0 | 11 |
| 35809.9 | 35809.5 | -11 | 25.80% | S | 33 | 28 | 0 | 11 |
| 36392.5 | 36392.1 | -11 | 25.80% | S | 33 | 28 | 0 | 13 |
| 35591.8 | 35592 | 6 | 25.70% | S | 32 | 27 | 3 | 10 |
| 35226.4 | 35226.4 | 0 | 25.70% | F2 | 29 | 24 | 0 | 14 |
| 35708.8 | 35708.6 | -6 | 25.00% | F1 | 32 | 27 | 2 | 11 |
| 33840.2 | 33840.5 | 9 | 25.00% | S | 30 | 25 | 0 | 8 |
| 35636.8 | 35637 | 6 | 24.70% | F1 | 33 | 28 | 1 | 10 |
| 35590.8 | 35591 | 6 | 24.60% | S | 32 | 27 | 1 | 11 |
| 36102.2 | 36102 | -6 | 24.10% | S | 33 | 28 | 2 | 11 |
| 37341.3 | 37341 | -8 | 23.50% | S | 34 | 29 | 2 | 14 |
| 36682.7 | 36683 | 8 | 23.10% | S | 31 | 26 | 3 | 15 |
| 34833.1 | 34833.5 | 11 | 22.90% | F1 | 30 | 25 | 3 | 10 |
| 36900.9 | 36901 | 3 | 22.40% | S | 32 | 27 | 0 | 16 |
| 35882 | 35882 | 0 | 22.30% | S | 32 | 27 | 1 | 12 |
| 35632.7 | 35633 | 8 | 22.10% | F1 | 29 | 24 | 3 | 14 |
| 34862.1 | 34862 | -3 | 22.00% | S | 32 | 27 | 0 | 9 |
| 34642.9 | 34643.1 | 6 | 21.80% | S | 31 | 26 | 1 | 9 |
| 36293.4 | 36293 | -11 | 21.70% | F1 | 34 | 29 | 1 | 11 |
| 36247.3 | 36247.5 | 6 | 21.50% | S | 33 | 28 | 1 | 12 |
| 34978.2 | 34978.5 | 9 | 20.70% | F1 | 30 | 25 | 2 | 11 |
| 36978 | 36977.9 | -3 | 20.40% | S | 35 | 30 | 1 | 12 |
| 36173.3 | 36173.5 | 6 | 20.40% | S | 32 | 27 | 1 | 13 |
| 34716 | 34716.5 | 14 | 19.90% | S | 30 | 25 | 4 | 9 |
| 35370.5 | 35370.5 | 0 | 19.50% | S | 31 | 26 | 0 | 12 |
| 36876.9 | 36877 | 3 | 19.30% | F1 | 34 | 29 | 3 | 12 |
| 36511.6 | 36511.1 | -14 | 19.30% | F1 | 33 | 28 | 3 | 12 |
| 34393.6 | 34393.4 | -6 | 19.20% | F1 | 28 | 23 | 3 | 11 |
| 35049.2 | 35049.5 | 9 | 18.90% | F1 | 29 | 24 | 1 | 13 |
| 35665.8 | 35665.5 | -8 | 18.30% | F2 | 31 | 26 | 0 | 13 |
| 34613.9 | 34613.5 | -12 | 18.30% | F1 | 29 | 24 | 4 | 10 |
| 36365.4 | 36365.5 | 3 | 17.90% | F1 | 33 | 28 | 2 | 12 |
| 34468.7 | 34468.5 | -6 | 17.80% | F1 | 31 | 26 | 0 | 9 |
| 35850.9 | 35851 | 3 | 17.40% | F1 | 30 | 25 | 0 | 15 |
| 36729.7 | 36730 | 8 | 17.10% | F1 | 34 | 29 | 0 | 13 |
| 35633.7 | 35633.3 | -11 | 17.10% | F1 | 31 | 26 | 0 | 13 |
| 35194.3 | 35194 | -9 | 16.90% | F1 | 29 | 24 | 0 | 14 |
| 35562.7 | 35562.5 | -6 | 16.50% | F1 | 32 | 27 | 1 | 11 |
| 36683.7 | 36683.4 | -8 | 16.40% | S | 33 | 28 | 0 | 14 |
| 34277.6 | 34278 | 12 | 15.40% | S | 30 | 25 | 1 | 9 |
| 35517.7 | 35517.5 | -6 | 15.30% | S | 31 | 26 | 3 | 11 |
| 34351.6 | 34351.4 | -6 | 15.00% | F2 | 27 | 22 | 3 | 12 |
| 37096.1 | 37096.9 | 22 | 14.40% | F1 | 35 | 30 | 2 | 12 |
| 34760 | 34760 | 0 | 14.30% | F1 | 31 | 26 | 0 | 10 |
| 33959.3 | 33959.5 | 6 | 14.10% | F1 | 30 | 25 | 3 | 7 |
| 35781.9 | 35781.5 | -11 | 13.60% | F1 | 33 | 28 | 0 | 11 |
| 35080.3 | 35080.5 | 6 | 13.40% | S | 31 | 26 | 2 | 10 |
| 33475.9 | 33475.5 | -12 | 12.40% | S | 29 | 24 | 2 | 7 |
| 35709.9 | 35710.6 | 20 | 12.10% | F1 | 32 | 27 | 4 | 10 |
| 37023 | 37023 | 0 | 11.70% | F1 | 34 | 29 | 4 | 12 |
| 33766.1 | 33766.5 | 12 | 11.50% | S | 29 | 24 | 0 | 9 |
| 33986.3 | 33986.6 | 9 | 10.70% | S | 30 | 25 | 1 | 8 |
| 34249.5 | 34250 | 15 | 9.90% | F1 | 30 | 25 | 1 | 9 |
| 35081.3 | 35081.1 | -6 | 9.70% | S | 31 | 26 | 4 | 9 |
| 34205.5 | 34205.6 | 3 | 9.60% | S | 31 | 26 | 0 | 8 |
| 34206.5 | 34206.5 | 0 | 9.60% | F2 | 27 | 22 | 4 | 11 |
| 36539.6 | 36539.5 | -3 | 9.50% | S | 33 | 28 | 3 | 12 |
| 33548.9 | 33549 | 3 | 9.30% | S | 30 | 25 | 0 | 7 |
| 33913.3 | 33913.5 | 6 | 9.10% | S | 29 | 24 | 3 | 8 |
| 33549.9 | 33549.5 | -12 | 9.10% | F2 | 26 | 21 | 4 | 10 |
| 36730.8 | 36730.5 | -8 | 8.60% | F1 | 34 | 29 | 2 | 12 |
| 34541.8 | 34542.1 | 9 | 8.40% | F1 | 30 | 25 | 3 | 9 |
| 34643.9 | 34644 | 3 | 8.30% | S | 31 | 26 | 3 | 8 |
| 34423.7 | 34424 | 9 | 8.30% | S | 30 | 25 | 2 | 9 |
| 35225.4 | 35225.9 | 14 | 8.20% | S | 31 | 26 | 1 | 11 |
| 34687.9 | 34687.6 | -9 | 8.20% | F1 | 30 | 25 | 4 | 9 |
| 34319.6 | 34319.2 | -12 | 6.90% | F1 | 27 | 22 | 3 | 12 |
| 34935.1 | 34935.1 | 0 | 6.70% | F2 | 29 | 24 | 0 | 13 |
| 34059.4 | 34059 | -12 | 6.50% | S | 29 | 24 | 4 | 8 |
| 37095.1 | 37095.5 | 11 | 6.40% | F1 | 35 | 30 | 0 | 13 |
| 34570.8 | 34570.6 | -6 | 6.10% | F2 | 28 | 23 | 2 | 12 |
| 36510.5 | 36511 | 14 | 5.90% | F1 | 33 | 28 | 1 | 13 |
| 35008.2 | 35008.5 | 9 | 5.80% | S | 32 | 27 | 1 | 9 |
| 36946.9 | 36947.4 | 14 | 5.60% | F1 | 33 | 28 | 0 | 15 |
| 37530.4 | 37530.5 | 3 | 5.60% | F1 | 33 | 28 | 2 | 16 |
| 33404.8 | 33404.5 | -9 | 5.60% | F2 | 28 | 23 | 0 | 9 |
| 34934.2 | 34934.5 | 9 | 5.30% | S | 31 | 26 | 1 | 10 |
| 33666 | 33666.5 | 15 | 5.20% | F1 | 28 | 23 | 4 | 8 |
| 34424.7 | 34424.5 | -6 | 5.00% | S | 30 | 25 | 4 | 8 |
| 33698.1 | 33698.5 | 12 | 4.70% | F2 | 28 | 23 | 4 | 8 |
| 37850.8 | 37851.2 | 11 | 4.70% | S | 33 | 28 | 4 | 16 |
| 36685.7 | 36685.5 | -5 | 4.70% | F2 | 31 | 26 | 1 | 16 |
| 33302.7 | 33302.6 | -3 | 4.60% | F1 | 29 | 24 | 3 | 6 |
| 34132.5 | 34132.5 | 0 | 4.60% | S | 30 | 25 | 2 | 8 |
| 36000.1 | 36000.5 | 11 | 4.50% | F1 | 32 | 27 | 2 | 12 |
| 34177.5 | 34177.6 | 3 | 4.50% | F1 | 31 | 26 | 0 | 8 |
| 33404.8 | 33404.5 | -9 | 4.40% | F2 | 28 | 23 | 0 | 9 |
| 38843.6 | 38844 | 10 | 4.40% | F1 | 35 | 30 | 2 | 18 |
| 34394.6 | 34394.5 | -3 | 4.40% | F1 | 30 | 25 | 0 | 10 |
| 33666 | 33666.5 | 15 | 4.20% | F1 | 28 | 23 | 4 | 8 |
| 37388.4 | 37388.5 | 3 | 4.20% | F1 | 35 | 30 | 4 | 12 |
| 33695 | 33695 | 0 | 4.00% | F2 | 26 | 21 | 3 | 11 |
| 36437.5 | 36437.5 | 0 | 3.60% | F1 | 32 | 27 | 3 | 13 |
| 34683.9 | 34684 | 3 | 3.60% | F1 | 28 | 23 | 1 | 13 |
| 34177.5 | 34177.6 | 3 | 3.50% | F1 | 31 | 26 | 0 | 8 |
| 34788 | 34788.3 | 9 | 2.70% | S | 31 | 26 | 0 | 10 |
| 37533.5 | 37533.6 | 3 | 2.70% | F1 | 35 | 30 | 3 | 13 |
| 37167.1 | 37167.5 | 11 | 2.60% | F1 | 34 | 29 | 1 | 14 |
| 37022 | 37021.7 | -8 | 2.40% | F1 | 34 | 29 | 2 | 13 |
| 35414.5 | 35414.5 | 0 | 2.40% | F1 | 30 | 25 | 1 | 13 |
| 37966.8 | 37966.5 | -8 | 2.20% | F1 | 33 | 28 | 1 | 18 |
| 37387.3 | 37387.4 | 3 | 2.10% | F1 | 35 | 30 | 2 | 13 |
| 37120.1 | 37119.3 | -22 | 1.70% | S | 31 | 26 | 4 | 16 |
| 36802.8 | 36803 | 5 | 1.60% | F1 | 33 | 28 | 3 | 13 |
| 37824.7 | 37825.5 | 21 | 1.50% | F1 | 35 | 30 | 3 | 14 |
| 36875.9 | 36876 | 3 | 1.30% | F1 | 34 | 29 | 1 | 13 |
| 36949 | 36948.5 | -14 | 1.30% | F1 | 33 | 28 | 4 | 13 |
| 33667 | 33667.6 | 18 | 1.30% | F1 | 30 | 25 | 1 | 7 |
| 37314.3 | 37313.9 | -11 | 1.20% | F1 | 34 | 29 | 4 | 13 |
| 37459.4 | 37460 | 16 | 1.00% | F1 | 34 | 29 | 3 | 14 |
| 37532.5 | 37532.5 | 0 | 0.80% | F1 | 35 | 30 | 1 | 14 |
| 33183.6 | 33184 | 12 | 0.80% | S | 29 | 24 | 0 | 7 |
| 34976.2 | 34975.6 | -17 | 0.60% | F1 | 28 | 23 | 3 | 13 |
| 37678.6 | 37678.5 | -3 | 0.50% | F1 | 35 | 30 | 2 | 14 |
| 36827.8 | 36828 | 5 | 0.50% | S | 31 | 26 | 2 | 16 |
| 33622 | 33622.5 | 15 | 0.50% | S | 29 | 24 | 3 | 7 |
| 34129.4 | 34128.7 | -21 | 0.40% | S | 28 | 23 | 1 | 11 |
| 37605.6 | 37606 | 11 | 0.40% | F1 | 34 | 29 | 4 | 14 |
| 33256.7 | 33257.1 | 12 | 0.40% | S | 28 | 23 | 3 | 7 |
| 37241.2 | 37241.5 | 8 | 0.40% | F1 | 35 | 30 | 1 | 13 |
| 34392.6 | 34392.6 | 0 | 0.30% | F1 | 28 | 23 | 1 | 12 |

^a^Relative intensity based on the intensities of the intact masses after deconvolution.

^b^total number of hexoses (Hex), N-acetylhexosamine (HexNAc), fucose (Fuc) and sialic acids (SA)

Table S8. Putatively assigned AGP glycoforms by nano-CZE-MS cut 3 (ordered by decreasing relative intensity).

| Theoretical Mass [Da] | Observed Mass [Da] | Deviation [ppm] | Rel. Int^a^ | Variant | Glycosylation^b^ | | | |
| --- | --- | --- | --- | --- | --- | --- | --- | --- |
|  |  |  |  |  | Hex | HexNac | Fuc | SA |
| 35635.8 | 35635.5 | -8 | 100.00% | F1 | 31 | 26 | 4 | 11 |
| 35927 | 35926.9 | -3 | 85.80% | F1 | 31 | 26 | 4 | 12 |
| 35198.4 | 35198.5 | 3 | 77.00% | F1 | 31 | 26 | 3 | 10 |
| 37239.2 | 37239.5 | 8 | 74.20% | F1 | 33 | 28 | 2 | 15 |
| 36145.2 | 36144.9 | -8 | 71.90% | F1 | 32 | 27 | 1 | 13 |
| 36249.3 | 36249.4 | 3 | 71.30% | F2 | 31 | 26 | 2 | 14 |
| 35592.7 | 35592.6 | -3 | 68.40% | F2 | 30 | 25 | 2 | 13 |
| 36072.2 | 36072.5 | 8 | 68.30% | F1 | 31 | 26 | 3 | 13 |
| 36655.7 | 36655.5 | -5 | 67.80% | F1 | 33 | 28 | 0 | 14 |
| 37020 | 37020.5 | 14 | 67.30% | F1 | 32 | 27 | 3 | 15 |
| 36436.5 | 36437 | 14 | 66.40% | F1 | 32 | 27 | 1 | 14 |
| 35884 | 35884.1 | 3 | 63.70% | F2 | 30 | 25 | 2 | 14 |
| 36976 | 36976.5 | 14 | 63.20% | S | 33 | 28 | 2 | 14 |
| 36363.4 | 36363.5 | 3 | 62.20% | F1 | 31 | 26 | 3 | 14 |
| 36541.6 | 36541.5 | -3 | 60.60% | F2 | 31 | 26 | 4 | 14 |
| 35958.1 | 35958.5 | 11 | 60.10% | F2 | 31 | 26 | 2 | 13 |
| 37385.3 | 37385.5 | 5 | 59.60% | F1 | 33 | 28 | 3 | 15 |
| 34615.9 | 34616 | 3 | 55.50% | F1 | 31 | 26 | 3 | 8 |
| 35737.9 | 35738 | 3 | 54.30% | S | 32 | 27 | 4 | 10 |
| 36540.6 | 36540.5 | -3 | 53.70% | F2 | 31 | 26 | 2 | 15 |
| 36585.6 | 36585.5 | -3 | 53.50% | F1 | 34 | 29 | 3 | 11 |
| 35270.5 | 35270.9 | 11 | 48.00% | F1 | 30 | 25 | 4 | 11 |
| 36582.6 | 36582.5 | -3 | 46.90% | F1 | 32 | 27 | 2 | 14 |
| 34644.9 | 34644.5 | -12 | 46.80% | F2 | 29 | 24 | 2 | 11 |
| 36031.1 | 36031 | -3 | 46.50% | F2 | 32 | 27 | 0 | 13 |
| 34906.1 | 34906.6 | 14 | 46.00% | F1 | 31 | 26 | 1 | 10 |
| 35011.3 | 35011 | -9 | 43.90% | F2 | 30 | 25 | 4 | 10 |
| 36220.3 | 36221 | 19 | 42.30% | F1 | 33 | 28 | 3 | 11 |
| 35375.6 | 35375.5 | -3 | 41.80% | F2 | 31 | 26 | 2 | 11 |
| 36395.5 | 36396 | 14 | 41.00% | F2 | 31 | 26 | 3 | 14 |
| 34686.9 | 34686.5 | -12 | 39.60% | F1 | 30 | 25 | 2 | 10 |
| 35446.7 | 35447.1 | 11 | 39.60% | S | 32 | 27 | 4 | 9 |
| 36611.7 | 36611.6 | -3 | 38.90% | S | 32 | 27 | 4 | 13 |
| 36104.2 | 36104.5 | 8 | 38.70% | F2 | 31 | 26 | 3 | 13 |
| 36979 | 36979.5 | 14 | 37.20% | F2 | 33 | 28 | 0 | 15 |
| 35738.9 | 35738.5 | -11 | 37.20% | F2 | 30 | 25 | 3 | 13 |
| 36902.9 | 36903 | 3 | 36.70% | S | 32 | 27 | 4 | 14 |
| 35854 | 35854.4 | 11 | 35.20% | F1 | 32 | 27 | 1 | 12 |
| 35445.6 | 35446 | 11 | 35.00% | S | 32 | 27 | 2 | 10 |
| 36219.3 | 36219.1 | -6 | 34.80% | F1 | 33 | 28 | 1 | 12 |
| 34496.8 | 34497.1 | 9 | 34.20% | S | 31 | 26 | 0 | 9 |
| 36657.7 | 36658.5 | 22 | 33.60% | F1 | 33 | 28 | 4 | 12 |
| 35123.3 | 35123.6 | 9 | 32.50% | F1 | 30 | 25 | 1 | 12 |
| 37093.1 | 37093.3 | 5 | 31.40% | F1 | 33 | 28 | 1 | 15 |
| 35999.1 | 35999 | -3 | 30.80% | F1 | 32 | 27 | 0 | 13 |
| 36873.9 | 36873.9 | 0 | 30.80% | F1 | 32 | 27 | 2 | 15 |
| 36290.3 | 36290 | -8 | 30.40% | F1 | 32 | 27 | 0 | 14 |
| 35780.9 | 35780.5 | -11 | 30.10% | F1 | 31 | 26 | 3 | 12 |
| 36727.7 | 36727.9 | 5 | 29.50% | F1 | 32 | 27 | 1 | 15 |
| 35883 | 35883 | 0 | 29.20% | S | 32 | 27 | 3 | 11 |
| 36321.4 | 36321.5 | 3 | 29.00% | S | 34 | 29 | 1 | 11 |
| 36613.6 | 36613.5 | -3 | 29.00% | F2 | 32 | 27 | 0 | 15 |
| 34395.7 | 34395.5 | -6 | 27.50% | F1 | 30 | 25 | 2 | 9 |
| 36464.5 | 36465 | 14 | 27.10% | S | 32 | 27 | 1 | 14 |
| 36950 | 36950.5 | 14 | 26.60% | F1 | 35 | 30 | 1 | 12 |
| 37677.6 | 37677.5 | -3 | 25.90% | F1 | 35 | 30 | 0 | 15 |
| 35226.4 | 35226.4 | 0 | 25.70% | F2 | 29 | 24 | 0 | 14 |
| 36757.8 | 36758 | 5 | 25.60% | S | 34 | 29 | 0 | 13 |
| 35372.6 | 35373 | 11 | 25.40% | S | 31 | 26 | 4 | 10 |
| 37271.3 | 37271.5 | 5 | 25.30% | F2 | 33 | 28 | 2 | 15 |
| 35707.8 | 35707.5 | -8 | 24.70% | F1 | 32 | 27 | 0 | 12 |
| 36174.3 | 36174.4 | 3 | 23.60% | S | 32 | 27 | 3 | 12 |
| 37603.5 | 37603.5 | 0 | 23.40% | F1 | 34 | 29 | 0 | 16 |
| 37126.1 | 37126.1 | 0 | 23.20% | F2 | 33 | 28 | 3 | 14 |
| 34833.1 | 34833.5 | 11 | 22.90% | F1 | 30 | 25 | 3 | 10 |
| 34862.1 | 34862 | -3 | 22.00% | S | 32 | 27 | 0 | 9 |
| 34642.9 | 34643.1 | 6 | 21.80% | S | 31 | 26 | 1 | 9 |
| 36293.4 | 36293 | -11 | 21.70% | F1 | 34 | 29 | 1 | 11 |
| 35156.4 | 35156.5 | 3 | 21.40% | F2 | 30 | 25 | 3 | 11 |
| 34978.2 | 34978.5 | 9 | 20.70% | F1 | 30 | 25 | 2 | 11 |
| 36978 | 36977.9 | -3 | 20.40% | S | 35 | 30 | 1 | 12 |
| 35158.4 | 35158.5 | 3 | 20.10% | F2 | 32 | 27 | 2 | 9 |
| 35450.7 | 35451.5 | 23 | 20.00% | F2 | 32 | 27 | 4 | 9 |
| 34716 | 34716.5 | 14 | 19.90% | S | 30 | 25 | 4 | 9 |
| 36829.9 | 36830 | 3 | 19.50% | S | 33 | 28 | 1 | 14 |
| 36319.4 | 36319.9 | 14 | 19.30% | S | 32 | 27 | 2 | 13 |
| 34789.1 | 34789 | -3 | 19.10% | S | 31 | 26 | 2 | 9 |
| 35665.8 | 35665.5 | -8 | 18.30% | F2 | 31 | 26 | 0 | 13 |
| 35154.4 | 35154 | -11 | 18.20% | S | 32 | 27 | 2 | 9 |
| 35052.3 | 35052 | -9 | 18.10% | F1 | 31 | 26 | 2 | 10 |
| 35739.9 | 35739.5 | -11 | 17.80% | F2 | 32 | 27 | 0 | 12 |
| 35741.9 | 35742.1 | 6 | 17.80% | F2 | 32 | 27 | 4 | 10 |
| 34468.7 | 34468.5 | -6 | 17.80% | F1 | 31 | 26 | 0 | 9 |
| 35815 | 35815 | 0 | 17.70% | F2 | 33 | 28 | 2 | 10 |
| 34104.4 | 34104 | -12 | 17.10% | F1 | 30 | 25 | 2 | 8 |
| 35633.7 | 35633.3 | -11 | 17.10% | F1 | 31 | 26 | 0 | 13 |
| 35155.4 | 35155.5 | 3 | 17.00% | F2 | 30 | 25 | 1 | 12 |
| 36106.2 | 36106 | -6 | 16.90% | F2 | 33 | 28 | 2 | 11 |
| 35562.7 | 35562.5 | -6 | 16.50% | F1 | 32 | 27 | 1 | 11 |
| 34865.1 | 34865.1 | 0 | 16.00% | F2 | 30 | 25 | 3 | 10 |
| 34791 | 34790.9 | -3 | 15.80% | F2 | 29 | 24 | 3 | 11 |
| 35125.3 | 35125.5 | 6 | 15.10% | F1 | 32 | 27 | 0 | 10 |
| 35594.8 | 35595.1 | 8 | 14.70% | F2 | 32 | 27 | 1 | 11 |
| 35522.7 | 35523.5 | 23 | 14.70% | F2 | 33 | 28 | 0 | 10 |
| 34760 | 34760 | 0 | 14.30% | F1 | 31 | 26 | 0 | 10 |
| 34715.9 | 34715.5 | -12 | 14.20% | F2 | 28 | 23 | 1 | 13 |
| 33959.3 | 33959.5 | 6 | 14.10% | F1 | 30 | 25 | 3 | 7 |
| 34323.6 | 34324.1 | 15 | 13.70% | F1 | 31 | 26 | 1 | 8 |
| 37124.1 | 37124 | -3 | 13.40% | F2 | 31 | 26 | 4 | 16 |
| 35521.7 | 35521.5 | -6 | 13.10% | F2 | 31 | 26 | 3 | 11 |
| 34979.2 | 34979.5 | 9 | 12.30% | F1 | 30 | 25 | 4 | 10 |
| 35416.6 | 35416.4 | -6 | 12.00% | F1 | 32 | 27 | 0 | 11 |
| 34250.6 | 34251 | 12 | 11.90% | F1 | 30 | 25 | 3 | 8 |
| 35300.5 | 35300.5 | 0 | 11.70% | S | 32 | 27 | 3 | 9 |
| 35301.5 | 35302 | 14 | 11.60% | F2 | 30 | 25 | 2 | 12 |
| 34794.1 | 34795 | 26 | 11.60% | F2 | 31 | 26 | 4 | 8 |
| 35664.8 | 35665.1 | 8 | 11.20% | S | 33 | 28 | 1 | 10 |
| 35959.1 | 35959 | -3 | 10.90% | F2 | 31 | 26 | 4 | 12 |
| 34501.8 | 34501.6 | -6 | 10.90% | F2 | 31 | 26 | 2 | 8 |
| 34646.9 | 34646.5 | -12 | 10.80% | F2 | 31 | 26 | 1 | 9 |
| 35489.6 | 35489.5 | -3 | 10.50% | F1 | 31 | 26 | 3 | 11 |
| 35811.9 | 35812.1 | 6 | 10.30% | F2 | 31 | 26 | 1 | 13 |
| 36250.4 | 36250.5 | 3 | 10.30% | F2 | 31 | 26 | 4 | 13 |
| 33813.2 | 33813.5 | 9 | 10.00% | F1 | 30 | 25 | 2 | 7 |
| 34249.5 | 34250 | 15 | 9.90% | F1 | 30 | 25 | 1 | 9 |
| 36397.5 | 36397.5 | 0 | 9.70% | F2 | 33 | 28 | 2 | 12 |
| 35081.3 | 35081.1 | -6 | 9.70% | S | 31 | 26 | 4 | 9 |
| 35591.7 | 35591.5 | -6 | 9.50% | F2 | 30 | 25 | 0 | 14 |
| 35082.3 | 35082.6 | 9 | 9.20% | F2 | 29 | 24 | 3 | 12 |
| 36396.5 | 36396.5 | 0 | 8.90% | F2 | 33 | 28 | 0 | 13 |
| 35373.6 | 35373.5 | -3 | 8.80% | F2 | 29 | 24 | 3 | 13 |
| 35488.6 | 35489 | 11 | 8.70% | F1 | 31 | 26 | 1 | 12 |
| 35374.5 | 35374.5 | 0 | 8.60% | F2 | 31 | 26 | 0 | 12 |
| 35886 | 35886.5 | 14 | 8.60% | F2 | 32 | 27 | 1 | 12 |
| 36033.2 | 36033 | -6 | 8.50% | F2 | 32 | 27 | 4 | 11 |
| 34541.8 | 34542.1 | 9 | 8.40% | F1 | 30 | 25 | 3 | 9 |
| 35230.5 | 35231 | 14 | 8.40% | F2 | 31 | 26 | 3 | 10 |
| 34687.9 | 34687.6 | -9 | 8.20% | F1 | 30 | 25 | 4 | 9 |
| 33447.8 | 33447.5 | -9 | 8.00% | F1 | 29 | 24 | 2 | 7 |
| 36030.1 | 36030 | -3 | 8.00% | F2 | 30 | 25 | 3 | 14 |
| 35226.5 | 35226.6 | 3 | 7.90% | S | 31 | 26 | 3 | 10 |
| 34645.9 | 34645.5 | -12 | 7.90% | F2 | 29 | 24 | 4 | 10 |
| 33958.3 | 33958.5 | 6 | 7.60% | F1 | 30 | 25 | 1 | 8 |
| 35009.2 | 35009.4 | 6 | 7.60% | F2 | 30 | 25 | 0 | 12 |
| 36324.4 | 36324.5 | 3 | 7.40% | F2 | 32 | 27 | 4 | 12 |
| 37196.1 | 37196 | -3 | 7.20% | F2 | 32 | 27 | 0 | 17 |
| 35813 | 35813 | 0 | 7.10% | F2 | 31 | 26 | 3 | 12 |
| 35519.7 | 35519.3 | -11 | 6.70% | F2 | 29 | 24 | 4 | 13 |
| 36471.6 | 36472.5 | 25 | 6.20% | F2 | 34 | 29 | 2 | 11 |
| 34570.8 | 34570.6 | -6 | 6.10% | F2 | 28 | 23 | 2 | 12 |
| 33520.9 | 33521 | 3 | 6.00% | F1 | 30 | 25 | 0 | 7 |
| 36510.5 | 36511 | 14 | 5.90% | F1 | 33 | 28 | 1 | 13 |
| 34935.2 | 34935.5 | 9 | 5.70% | S | 31 | 26 | 3 | 9 |
| 36177.3 | 36177.5 | 6 | 5.60% | F2 | 32 | 27 | 1 | 13 |
| 34031.4 | 34032 | 18 | 5.30% | F1 | 29 | 24 | 4 | 8 |
| 34424.7 | 34424.5 | -6 | 5.00% | S | 30 | 25 | 4 | 8 |
| 33739.1 | 33739.5 | 12 | 4.70% | F1 | 29 | 24 | 2 | 8 |
| 36946.9 | 36947.4 | 14 | 4.50% | F1 | 33 | 28 | 0 | 15 |
| 37530.4 | 37530.5 | 3 | 4.50% | F1 | 33 | 28 | 2 | 16 |
| 36686.7 | 36687 | 8 | 4.30% | F2 | 31 | 26 | 3 | 15 |
| 33666 | 33666.5 | 15 | 4.20% | F1 | 28 | 23 | 4 | 8 |
| 37388.4 | 37388.5 | 3 | 4.20% | F1 | 35 | 30 | 4 | 12 |
| 36437.5 | 36437.5 | 0 | 3.60% | F1 | 32 | 27 | 3 | 13 |
| 36178.3 | 36178.5 | 6 | 3.60% | F2 | 32 | 27 | 3 | 12 |
| 34177.5 | 34177.6 | 3 | 3.50% | F1 | 31 | 26 | 0 | 8 |
| 36762.8 | 36762.6 | -5 | 3.50% | F2 | 34 | 29 | 2 | 12 |
| 34105.4 | 34105 | -12 | 3.50% | F1 | 30 | 25 | 4 | 7 |
| 34394.6 | 34394.5 | -3 | 3.50% | F1 | 30 | 25 | 0 | 10 |
| 34130.5 | 34130 | -15 | 3.40% | S | 28 | 23 | 3 | 10 |
| 37342.3 | 37342 | -8 | 3.40% | F2 | 32 | 27 | 1 | 17 |
| 37168.2 | 37168 | -5 | 3.20% | F1 | 34 | 29 | 3 | 13 |
| 36469.6 | 36469.2 | -11 | 3.00% | F2 | 32 | 27 | 3 | 13 |
| 36835.9 | 36835.5 | -11 | 2.90% | F2 | 35 | 30 | 0 | 12 |
| 37167.1 | 37167.5 | 11 | 2.60% | F1 | 34 | 29 | 1 | 14 |
| 33838.2 | 33838.7 | 15 | 2.50% | S | 28 | 23 | 1 | 10 |
| 37022 | 37021.7 | -8 | 2.40% | F1 | 34 | 29 | 2 | 13 |
| 36543.6 | 36544 | 11 | 2.40% | F2 | 33 | 28 | 3 | 12 |
| 33155.5 | 33155.9 | 12 | 2.20% | F1 | 29 | 24 | 0 | 7 |
| 36687.7 | 36688 | 8 | 2.00% | F2 | 33 | 28 | 0 | 14 |
| 36688.7 | 36689 | 8 | 1.60% | F2 | 33 | 28 | 2 | 13 |
| 36802.8 | 36803 | 5 | 1.60% | F1 | 33 | 28 | 3 | 13 |
| 36875.9 | 36876 | 3 | 1.30% | F1 | 34 | 29 | 1 | 13 |
| 37128.2 | 37128.1 | -3 | 1.20% | F2 | 35 | 30 | 2 | 12 |
| 37314.3 | 37313.9 | -11 | 1.20% | F1 | 34 | 29 | 4 | 13 |
| 37240.2 | 37240.5 | 8 | 1.00% | F1 | 33 | 28 | 4 | 14 |
| 37459.4 | 37460 | 16 | 1.00% | F1 | 34 | 29 | 3 | 14 |
| 33301.7 | 33302 | 9 | 1.00% | F1 | 29 | 24 | 1 | 7 |
| 37532.5 | 37532.5 | 0 | 0.80% | F1 | 35 | 30 | 1 | 14 |
| 36833.9 | 36833.5 | -11 | 0.80% | F2 | 33 | 28 | 1 | 14 |
| 37054.1 | 37054.5 | 11 | 0.80% | F2 | 34 | 29 | 2 | 13 |
| 35925 | 35925.5 | 14 | 0.70% | F1 | 31 | 26 | 0 | 14 |
| 33374.8 | 33374.6 | -6 | 0.60% | F1 | 28 | 23 | 4 | 7 |
| 36907 | 36906.5 | -14 | 0.60% | F2 | 32 | 27 | 4 | 14 |
| 36759.8 | 36759.9 | 3 | 0.50% | S | 34 | 29 | 4 | 11 |
| 37420.4 | 37421 | 16 | 0.50% | F2 | 35 | 30 | 4 | 12 |
| 37605.6 | 37606 | 11 | 0.40% | F1 | 34 | 29 | 4 | 14 |
| 37122.1 | 37122.5 | 11 | 0.30% | S | 33 | 28 | 3 | 14 |
| 37272.3 | 37271.9 | -11 | 0.20% | F2 | 33 | 28 | 4 | 14 |
| 37781.7 | 37782.1 | 11 | 0.10% | F2 | 34 | 29 | 1 | 16 |
| 37313.3 | 37313 | -8 | 0.10% | F1 | 34 | 29 | 2 | 14 |
| 37750.7 | 37751.4 | 19 | 0.10% | F1 | 34 | 29 | 3 | 15 |
| 37970.9 | 37971.5 | 16 | 0.00% | F1 | 35 | 30 | 4 | 14 |
| 37823.7 | 37823.6 | -3 | 0.00% | F1 | 35 | 30 | 1 | 15 |
| 37310.2 | 37310 | -5 | 0.00% | F1 | 32 | 27 | 1 | 17 |

^a^Relative intensity based on the intensities of the intact masses after deconvolution.

^b^total number of hexoses (Hex), N-acetylhexosamine (HexNAc), fucose (Fuc) and sialic acids (SA)

Table S9. Putatively assigned AGP glycoforms from 1:20 diluted DBS eluate by nano-CZE-MS cut 1 (ordered by decreasing relative intensity).

| Theoretical Mass [Da] | Observed Mass [Da] | Deviation [ppm] | Rel. Int^a^ | Variant | Glycosylation^b^ | | | |
| --- | --- | --- | --- | --- | --- | --- | --- | --- |
|  |  |  |  |  | Hex | HexNac | Fuc | SA |
| 34131.4 | 34131.5 | 3 | 100.00% | S | 30 | 25 | 0 | 9 |
| 36320.4 | 36320.6 | 6 | 99.90% | S | 32 | 27 | 4 | 12 |
| 35153.4 | 35153.9 | 14 | 98.40% | S | 32 | 27 | 0 | 10 |
| 35663.8 | 35664 | 6 | 81.40% | S | 31 | 26 | 4 | 11 |
| 35956.1 | 35956 | -3 | 76.80% | S | 33 | 28 | 1 | 11 |
| 35371.6 | 35372 | 11 | 73.90% | S | 31 | 26 | 2 | 11 |
| 35299.5 | 35299.5 | 0 | 58.80% | S | 32 | 27 | 1 | 10 |
| 35444.6 | 35445 | 11 | 57.40% | S | 32 | 27 | 0 | 11 |
| 36219.3 | 36219.1 | -6 | 52.00% | F1 | 33 | 28 | 1 | 12 |
| 36101.2 | 36101.1 | -3 | 51.70% | S | 33 | 28 | 0 | 12 |
| 34496.8 | 34497.1 | 9 | 51.20% | S | 31 | 26 | 0 | 9 |
| 35735.9 | 35735.5 | -11 | 47.60% | S | 32 | 27 | 0 | 12 |
| 35883 | 35883 | 0 | 43.70% | S | 32 | 27 | 3 | 11 |
| 34422.7 | 34423 | 9 | 43.50% | S | 30 | 25 | 0 | 10 |
| 35809.9 | 35809.5 | -11 | 38.60% | S | 33 | 28 | 0 | 11 |
| 33840.2 | 33840.5 | 9 | 37.40% | S | 30 | 25 | 0 | 8 |
| 35590.8 | 35591 | 6 | 36.80% | S | 32 | 27 | 1 | 11 |
| 36174.3 | 36174.4 | 3 | 35.30% | S | 32 | 27 | 3 | 12 |
| 34642.9 | 34643.1 | 6 | 32.70% | S | 31 | 26 | 1 | 9 |
| 34716 | 34716.5 | 14 | 29.80% | S | 30 | 25 | 4 | 9 |
| 34717 | 34717.5 | 14 | 28.70% | F2 | 28 | 23 | 3 | 12 |
| 34789.1 | 34789 | -3 | 28.60% | S | 31 | 26 | 2 | 9 |
| 35006.2 | 35006 | -6 | 28.20% | S | 30 | 25 | 2 | 11 |
| 33474.9 | 33475 | 3 | 26.60% | S | 29 | 24 | 0 | 8 |
| 33770.1 | 33770 | -3 | 24.90% | F2 | 29 | 24 | 0 | 9 |
| 36248.4 | 36248.5 | 3 | 24.30% | S | 33 | 28 | 3 | 11 |
| 34277.6 | 34278 | 12 | 23.10% | S | 30 | 25 | 1 | 9 |
| 35517.7 | 35517.5 | -6 | 22.90% | S | 31 | 26 | 3 | 11 |
| 34057.4 | 34057.5 | 3 | 22.80% | S | 29 | 24 | 0 | 10 |
| 34351.6 | 34351.4 | -6 | 22.50% | F2 | 27 | 22 | 3 | 12 |
| 35080.3 | 35080.5 | 6 | 20.10% | S | 31 | 26 | 2 | 10 |
| 33766.1 | 33766.5 | 12 | 17.10% | S | 29 | 24 | 0 | 9 |
| 33986.3 | 33986.6 | 9 | 15.90% | S | 30 | 25 | 1 | 8 |
| 34205.5 | 34205.6 | 3 | 14.40% | S | 31 | 26 | 0 | 8 |
| 36539.6 | 36539.5 | -3 | 14.20% | S | 33 | 28 | 3 | 12 |
| 33548.9 | 33549 | 3 | 13.90% | S | 30 | 25 | 0 | 7 |
| 34027.3 | 34026.7 | -18 | 13.70% | F1 | 27 | 22 | 1 | 12 |
| 34715 | 34715 | 0 | 13.60% | S | 30 | 25 | 2 | 10 |
| 33549.9 | 33549.5 | -12 | 13.50% | F2 | 26 | 21 | 4 | 10 |
| 34278.6 | 34279.1 | 15 | 12.50% | S | 30 | 25 | 3 | 8 |
| 34423.7 | 34424 | 9 | 12.30% | S | 30 | 25 | 2 | 9 |
| 35225.4 | 35225.9 | 14 | 12.20% | S | 31 | 26 | 1 | 11 |
| 36029.1 | 36029 | -3 | 9.90% | F2 | 30 | 25 | 1 | 15 |
| 33914.2 | 33914.4 | 6 | 9.20% | F2 | 27 | 22 | 2 | 11 |
| 34860.1 | 34860.5 | 11 | 9.20% | S | 30 | 25 | 1 | 11 |
| 33185.6 | 33185.5 | -3 | 9.20% | F2 | 27 | 22 | 1 | 9 |
| 34570.8 | 34570.6 | -6 | 9.10% | F2 | 28 | 23 | 2 | 12 |
| 34934.2 | 34934.5 | 9 | 8.00% | S | 31 | 26 | 1 | 10 |
| 32967.4 | 32967 | -12 | 8.00% | F2 | 26 | 21 | 4 | 8 |
| 34424.7 | 34424.5 | -6 | 7.50% | S | 30 | 25 | 4 | 8 |
| 32601.1 | 32601.6 | 15 | 6.60% | F2 | 25 | 20 | 2 | 9 |
| 32164.7 | 32164.5 | -6 | 6.50% | F2 | 25 | 20 | 3 | 7 |
| 33405.8 | 33405.4 | -12 | 5.10% | F2 | 28 | 23 | 2 | 8 |
| 34788 | 34788.3 | 9 | 4.10% | S | 31 | 26 | 0 | 10 |
| 33332.8 | 33333 | 6 | 3.10% | F2 | 27 | 22 | 4 | 8 |
| 33040.5 | 33041 | 15 | 2.40% | F2 | 27 | 22 | 2 | 8 |
| 33186.6 | 33186.5 | -3 | 1.80% | F2 | 27 | 22 | 3 | 8 |
| 32676.2 | 32676 | -6 | 1.80% | F2 | 26 | 21 | 4 | 7 |
| 33113.5 | 33114 | 15 | 1.70% | F2 | 28 | 23 | 0 | 8 |
| 32310.8 | 32311 | 6 | 1.10% | F2 | 25 | 20 | 4 | 7 |
| 34131.4 | 34131.5 | 3 | 100.00% | S | 30 | 25 | 0 | 9 |
| 36320.4 | 36320.6 | 6 | 99.90% | S | 32 | 27 | 4 | 12 |
| 35153.4 | 35153.9 | 14 | 98.40% | S | 32 | 27 | 0 | 10 |
| 35663.8 | 35664 | 6 | 81.40% | S | 31 | 26 | 4 | 11 |
| 35956.1 | 35956 | -3 | 76.80% | S | 33 | 28 | 1 | 11 |
| 35371.6 | 35372 | 11 | 73.90% | S | 31 | 26 | 2 | 11 |
| 35299.5 | 35299.5 | 0 | 58.80% | S | 32 | 27 | 1 | 10 |
| 35444.6 | 35445 | 11 | 57.40% | S | 32 | 27 | 0 | 11 |
| 36219.3 | 36219.1 | -6 | 52.00% | F1 | 33 | 28 | 1 | 12 |
| 36101.2 | 36101.1 | -3 | 51.70% | S | 33 | 28 | 0 | 12 |
| 34496.8 | 34497.1 | 9 | 51.20% | S | 31 | 26 | 0 | 9 |
| 35735.9 | 35735.5 | -11 | 47.60% | S | 32 | 27 | 0 | 12 |
| 35883 | 35883 | 0 | 43.70% | S | 32 | 27 | 3 | 11 |
| 34422.7 | 34423 | 9 | 43.50% | S | 30 | 25 | 0 | 10 |
| 35809.9 | 35809.5 | -11 | 38.60% | S | 33 | 28 | 0 | 11 |
| 33840.2 | 33840.5 | 9 | 37.40% | S | 30 | 25 | 0 | 8 |
| 35590.8 | 35591 | 6 | 36.80% | S | 32 | 27 | 1 | 11 |
| 36174.3 | 36174.4 | 3 | 35.30% | S | 32 | 27 | 3 | 12 |
| 34642.9 | 34643.1 | 6 | 32.70% | S | 31 | 26 | 1 | 9 |
| 34716 | 34716.5 | 14 | 29.80% | S | 30 | 25 | 4 | 9 |
| 34717 | 34717.5 | 14 | 28.70% | F2 | 28 | 23 | 3 | 12 |
| 34789.1 | 34789 | -3 | 28.60% | S | 31 | 26 | 2 | 9 |
| 35006.2 | 35006 | -6 | 28.20% | S | 30 | 25 | 2 | 11 |
| 33474.9 | 33475 | 3 | 26.60% | S | 29 | 24 | 0 | 8 |
| 33770.1 | 33770 | -3 | 24.90% | F2 | 29 | 24 | 0 | 9 |
| 36248.4 | 36248.5 | 3 | 24.30% | S | 33 | 28 | 3 | 11 |
| 34277.6 | 34278 | 12 | 23.10% | S | 30 | 25 | 1 | 9 |
| 35517.7 | 35517.5 | -6 | 22.90% | S | 31 | 26 | 3 | 11 |
| 34057.4 | 34057.5 | 3 | 22.80% | S | 29 | 24 | 0 | 10 |
| 34351.6 | 34351.4 | -6 | 22.50% | F2 | 27 | 22 | 3 | 12 |
| 35080.3 | 35080.5 | 6 | 20.10% | S | 31 | 26 | 2 | 10 |
| 33766.1 | 33766.5 | 12 | 17.10% | S | 29 | 24 | 0 | 9 |
| 33986.3 | 33986.6 | 9 | 15.90% | S | 30 | 25 | 1 | 8 |
| 34205.5 | 34205.6 | 3 | 14.40% | S | 31 | 26 | 0 | 8 |
| 36539.6 | 36539.5 | -3 | 14.20% | S | 33 | 28 | 3 | 12 |
| 33548.9 | 33549 | 3 | 13.90% | S | 30 | 25 | 0 | 7 |
| 34027.3 | 34026.7 | -18 | 13.70% | F1 | 27 | 22 | 1 | 12 |
| 34715 | 34715 | 0 | 13.60% | S | 30 | 25 | 2 | 10 |
| 33549.9 | 33549.5 | -12 | 13.50% | F2 | 26 | 21 | 4 | 10 |
| 34278.6 | 34279.1 | 15 | 12.50% | S | 30 | 25 | 3 | 8 |
| 34423.7 | 34424 | 9 | 12.30% | S | 30 | 25 | 2 | 9 |
| 35225.4 | 35225.9 | 14 | 12.20% | S | 31 | 26 | 1 | 11 |
| 36029.1 | 36029 | -3 | 9.90% | F2 | 30 | 25 | 1 | 15 |
| 33914.2 | 33914.4 | 6 | 9.20% | F2 | 27 | 22 | 2 | 11 |
| 34860.1 | 34860.5 | 11 | 9.20% | S | 30 | 25 | 1 | 11 |
| 33185.6 | 33185.5 | -3 | 9.20% | F2 | 27 | 22 | 1 | 9 |
| 34570.8 | 34570.6 | -6 | 9.10% | F2 | 28 | 23 | 2 | 12 |
| 34934.2 | 34934.5 | 9 | 8.00% | S | 31 | 26 | 1 | 10 |
| 32967.4 | 32967 | -12 | 8.00% | F2 | 26 | 21 | 4 | 8 |
| 34424.7 | 34424.5 | -6 | 7.50% | S | 30 | 25 | 4 | 8 |
| 32601.1 | 32601.6 | 15 | 6.60% | F2 | 25 | 20 | 2 | 9 |
| 32164.7 | 32164.5 | -6 | 6.50% | F2 | 25 | 20 | 3 | 7 |
| 33405.8 | 33405.4 | -12 | 5.10% | F2 | 28 | 23 | 2 | 8 |
| 34788 | 34788.3 | 9 | 4.10% | S | 31 | 26 | 0 | 10 |
| 33332.8 | 33333 | 6 | 3.10% | F2 | 27 | 22 | 4 | 8 |
| 33040.5 | 33041 | 15 | 2.40% | F2 | 27 | 22 | 2 | 8 |
| 33186.6 | 33186.5 | -3 | 1.80% | F2 | 27 | 22 | 3 | 8 |
| 32676.2 | 32676 | -6 | 1.80% | F2 | 26 | 21 | 4 | 7 |
| 33113.5 | 33114 | 15 | 1.70% | F2 | 28 | 23 | 0 | 8 |
| 32310.8 | 32311 | 6 | 1.10% | F2 | 25 | 20 | 4 | 7 |
| 33186.6 | 33186.5 | -3 | 1.80% | F2 | 27 | 22 | 3 | 8 |
| 32676.2 | 32676 | -6 | 1.80% | F2 | 26 | 21 | 4 | 7 |
| 33113.5 | 33114 | 15 | 1.70% | F2 | 28 | 23 | 0 | 8 |
| 32310.8 | 32311 | 6 | 1.10% | F2 | 25 | 20 | 4 | 7 |
| 33186.6 | 33186.5 | -3 | 1.80% | F2 | 27 | 22 | 3 | 8 |
| 32676.2 | 32676 | -6 | 1.80% | F2 | 26 | 21 | 4 | 7 |
| 33113.5 | 33114 | 15 | 1.70% | F2 | 28 | 23 | 0 | 8 |
| 32310.8 | 32311 | 6 | 1.10% | F2 | 25 | 20 | 4 | 7 |
| 33186.6 | 33186.5 | -3 | 1.80% | F2 | 27 | 22 | 3 | 8 |

^a^Relative intensity based on the intensities of the intact masses after deconvolution.

^b^total number of hexoses (Hex), N-acetylhexosamine (HexNAc), fucose (Fuc) and sialic acids (SA)

Table S10. Putatively assigned AGP glycoforms by nano-CZE-MS cut 3 (ordered by decreasing relative intensity).

| Theoretical Mass [Da] | Observed Mass [Da] | Deviation [ppm] | Rel. Int^a^ | Variant | Glycosylation^b^ | | | |
| --- | --- | --- | --- | --- | --- | --- | --- | --- |
|  |  |  |  |  | Hex | HexNac | Fuc | SA |
| 36074.2 | 36074.4 | 6 | 100.00% | F1 | 33 | 28 | 2 | 11 |
| 35490.6 | 35490.5 | -3 | 82.90% | F1 | 33 | 28 | 0 | 10 |
| 35928 | 35927.9 | -3 | 82.40% | F1 | 33 | 28 | 1 | 11 |
| 36249.3 | 36249.4 | 3 | 64.50% | F2 | 31 | 26 | 2 | 14 |
| 35929.1 | 35929 | -3 | 62.80% | F1 | 33 | 28 | 3 | 10 |
| 36320.4 | 36320.6 | 6 | 60.50% | S | 32 | 27 | 4 | 12 |
| 35153.4 | 35153.9 | 14 | 59.60% | S | 32 | 27 | 0 | 10 |
| 35998.1 | 35998.5 | 11 | 58.90% | F1 | 30 | 25 | 3 | 14 |
| 36584.6 | 36585 | 11 | 57.90% | F1 | 34 | 29 | 1 | 12 |
| 35884 | 35884.1 | 3 | 57.60% | F2 | 30 | 25 | 2 | 14 |
| 35958.1 | 35958.5 | 11 | 54.40% | F2 | 31 | 26 | 2 | 13 |
| 34980.2 | 34981.1 | 26 | 53.30% | F1 | 32 | 27 | 1 | 9 |
| 36071.1 | 36071.5 | 11 | 52.70% | F1 | 31 | 26 | 1 | 14 |
| 36001.1 | 36001.5 | 11 | 50.30% | F1 | 32 | 27 | 4 | 11 |
| 35446.6 | 35446.5 | -3 | 49.80% | F2 | 30 | 25 | 1 | 13 |
| 35663.8 | 35664 | 6 | 49.30% | S | 31 | 26 | 4 | 11 |
| 35737.9 | 35738 | 3 | 49.10% | S | 32 | 27 | 4 | 10 |
| 36540.6 | 36540.5 | -3 | 48.60% | F2 | 31 | 26 | 2 | 15 |
| 36175.3 | 36175.5 | 6 | 46.30% | S | 34 | 29 | 0 | 11 |
| 35666.8 | 35667 | 6 | 46.30% | F2 | 31 | 26 | 2 | 12 |
| 35853 | 35853.5 | 14 | 46.10% | F1 | 30 | 25 | 4 | 13 |
| 35269.4 | 35269.1 | -9 | 45.80% | F1 | 30 | 25 | 2 | 12 |
| 34644.9 | 34644.5 | -12 | 42.30% | F2 | 29 | 24 | 2 | 11 |
| 36103.2 | 36103 | -6 | 42.10% | F2 | 31 | 26 | 1 | 14 |
| 34936.2 | 34936 | -6 | 41.10% | F2 | 29 | 24 | 2 | 12 |
| 36465.5 | 36466 | 14 | 40.20% | S | 32 | 27 | 3 | 13 |
| 36612.7 | 36613 | 8 | 40.10% | S | 34 | 29 | 1 | 12 |
| 35486.6 | 35487 | 11 | 39.80% | F1 | 29 | 24 | 2 | 14 |
| 36220.3 | 36221 | 19 | 38.30% | F1 | 33 | 28 | 3 | 11 |
| 36394.5 | 36394.5 | 0 | 38.20% | S | 33 | 28 | 4 | 11 |
| 35563.7 | 35563.5 | -6 | 37.90% | F1 | 32 | 27 | 3 | 10 |
| 36395.5 | 36396 | 14 | 37.10% | F2 | 31 | 26 | 3 | 14 |
| 35272.5 | 35272 | -14 | 36.80% | F1 | 32 | 27 | 3 | 9 |
| 36656.7 | 36656.5 | -5 | 36.60% | F1 | 33 | 28 | 2 | 13 |
| 36538.6 | 36539 | 11 | 34.00% | S | 33 | 28 | 1 | 13 |
| 35738.9 | 35738.5 | -11 | 33.60% | F2 | 30 | 25 | 3 | 13 |
| 35957.1 | 35957.5 | 11 | 32.60% | S | 33 | 28 | 3 | 10 |
| 36147.2 | 36147 | -6 | 29.40% | F1 | 34 | 29 | 0 | 11 |
| 34719 | 34719.4 | 12 | 28.80% | F2 | 30 | 25 | 2 | 10 |
| 35999.1 | 35999 | -3 | 27.90% | F1 | 32 | 27 | 0 | 13 |
| 36290.3 | 36290 | -8 | 27.50% | F1 | 32 | 27 | 0 | 14 |
| 35780.9 | 35780.5 | -11 | 27.30% | F1 | 31 | 26 | 3 | 12 |
| 35122.3 | 35121.5 | -23 | 27.10% | F1 | 28 | 23 | 4 | 13 |
| 35883 | 35883 | 0 | 26.40% | S | 32 | 27 | 3 | 11 |
| 36321.4 | 36321.5 | 3 | 26.30% | S | 34 | 29 | 1 | 11 |
| 34907.1 | 34907.6 | 14 | 26.20% | F1 | 31 | 26 | 3 | 9 |
| 36103.3 | 36103.5 | 6 | 25.50% | S | 33 | 28 | 4 | 10 |
| 33988.3 | 33988.4 | 3 | 25.00% | F2 | 28 | 23 | 2 | 10 |
| 35124.3 | 35124 | -9 | 24.80% | F1 | 30 | 25 | 3 | 11 |
| 34612.8 | 34612.5 | -9 | 24.10% | F1 | 29 | 24 | 2 | 11 |
| 35809.9 | 35809.5 | -11 | 23.40% | S | 33 | 28 | 0 | 11 |
| 35591.8 | 35592 | 6 | 23.30% | S | 32 | 27 | 3 | 10 |
| 35372.5 | 35372.1 | -11 | 23.10% | F2 | 29 | 24 | 1 | 14 |
| 35708.8 | 35708.6 | -6 | 22.70% | F1 | 32 | 27 | 2 | 11 |
| 35636.8 | 35637 | 6 | 22.30% | F1 | 33 | 28 | 1 | 10 |
| 35590.8 | 35591 | 6 | 22.30% | S | 32 | 27 | 1 | 11 |
| 33955.2 | 33955.6 | 12 | 22.00% | F1 | 28 | 23 | 0 | 11 |
| 34498.8 | 34499.1 | 9 | 21.50% | F2 | 29 | 24 | 1 | 11 |
| 36174.3 | 36174.4 | 3 | 21.40% | S | 32 | 27 | 3 | 12 |
| 35882 | 35882 | 0 | 20.20% | S | 32 | 27 | 1 | 12 |
| 35632.7 | 35633 | 8 | 20.00% | F1 | 29 | 24 | 3 | 14 |
| 34862.1 | 34862 | -3 | 19.90% | S | 32 | 27 | 0 | 9 |
| 36293.4 | 36293 | -11 | 19.60% | F1 | 34 | 29 | 1 | 11 |
| 34758 | 34758.5 | 14 | 19.50% | F1 | 29 | 24 | 1 | 12 |
| 35855 | 35855.5 | 14 | 18.90% | F1 | 32 | 27 | 3 | 11 |
| 36511.6 | 36511.1 | -14 | 17.50% | F1 | 33 | 28 | 3 | 12 |
| 34717 | 34717.5 | 14 | 17.30% | F2 | 28 | 23 | 3 | 12 |
| 34789.1 | 34789 | -3 | 17.30% | S | 31 | 26 | 2 | 9 |
| 34834 | 34834 | 0 | 16.70% | F1 | 32 | 27 | 0 | 9 |
| 35052.3 | 35052 | -9 | 16.40% | F1 | 31 | 26 | 2 | 10 |
| 36365.4 | 36365.5 | 3 | 16.20% | F1 | 33 | 28 | 2 | 12 |
| 34133.4 | 34133 | -12 | 15.50% | F2 | 28 | 23 | 1 | 11 |
| 35194.3 | 35194 | -9 | 15.30% | F1 | 29 | 24 | 0 | 14 |
| 35227.4 | 35227.5 | 3 | 15.20% | F2 | 29 | 24 | 2 | 13 |
| 33770.1 | 33770 | -3 | 15.10% | F2 | 29 | 24 | 0 | 9 |
| 35518.7 | 35518.6 | -3 | 15.10% | S | 33 | 28 | 0 | 10 |
| 35562.7 | 35562.5 | -6 | 14.90% | F1 | 32 | 27 | 1 | 11 |
| 35340.5 | 35340.4 | -3 | 14.90% | F1 | 29 | 24 | 1 | 14 |
| 36248.4 | 36248.5 | 3 | 14.70% | S | 33 | 28 | 3 | 11 |
| 33259.7 | 33259.5 | -6 | 14.40% | F2 | 28 | 23 | 1 | 8 |
| 34614.9 | 34614.9 | 0 | 14.40% | F1 | 31 | 26 | 1 | 9 |
| 33624 | 33624.5 | 15 | 13.90% | F2 | 27 | 22 | 4 | 9 |
| 33590.9 | 33591 | 3 | 13.20% | F1 | 27 | 22 | 2 | 10 |
| 34715.9 | 34715.5 | -12 | 12.80% | F2 | 28 | 23 | 1 | 13 |
| 33841.2 | 33841.5 | 9 | 12.10% | S | 30 | 25 | 2 | 7 |
| 33475.9 | 33475.5 | -12 | 11.20% | S | 29 | 24 | 2 | 7 |
| 34469.7 | 34469.5 | -6 | 11.10% | F1 | 31 | 26 | 2 | 8 |
| 35416.6 | 35416.4 | -6 | 10.90% | F1 | 32 | 27 | 0 | 11 |
| 34250.6 | 34251 | 12 | 10.80% | F1 | 30 | 25 | 3 | 8 |
| 35300.5 | 35300.5 | 0 | 10.60% | S | 32 | 27 | 3 | 9 |
| 35341.5 | 35341.5 | 0 | 10.30% | F1 | 29 | 24 | 3 | 13 |
| 35664.8 | 35665.1 | 8 | 10.10% | S | 33 | 28 | 1 | 10 |
| 34611.8 | 34612 | 6 | 9.10% | F1 | 29 | 24 | 0 | 12 |
| 35081.3 | 35081.1 | -6 | 8.80% | S | 31 | 26 | 4 | 9 |
| 34206.5 | 34206.5 | 0 | 8.70% | F2 | 27 | 22 | 4 | 11 |
| 36539.6 | 36539.5 | -3 | 8.60% | S | 33 | 28 | 3 | 12 |
| 34497.8 | 34497.6 | -6 | 8.20% | S | 31 | 26 | 2 | 8 |
| 33549.9 | 33549.5 | -12 | 8.20% | F2 | 26 | 21 | 4 | 10 |
| 32968.4 | 32968 | -12 | 8.20% | F2 | 28 | 23 | 1 | 7 |
| 33083.5 | 33084.4 | 27 | 7.90% | F1 | 28 | 23 | 4 | 6 |
| 33885.2 | 33885 | -6 | 7.90% | F1 | 29 | 24 | 3 | 8 |
| 34278.6 | 34279.1 | 15 | 7.60% | S | 30 | 25 | 3 | 8 |
| 34643.9 | 34644 | 3 | 7.50% | S | 31 | 26 | 3 | 8 |
| 34423.7 | 34424 | 9 | 7.50% | S | 30 | 25 | 2 | 9 |
| 34687.9 | 34687.6 | -9 | 7.40% | F1 | 30 | 25 | 4 | 9 |
| 36030.1 | 36030 | -3 | 7.20% | F2 | 30 | 25 | 3 | 14 |
| 33736 | 33736.5 | 15 | 7.10% | F1 | 27 | 22 | 1 | 11 |
| 35009.2 | 35009.4 | 6 | 6.90% | F2 | 30 | 25 | 0 | 12 |
| 34718 | 34718 | 0 | 6.70% | F2 | 30 | 25 | 0 | 11 |
| 33767.1 | 33767.5 | 12 | 6.50% | S | 29 | 24 | 2 | 8 |
| 34101.4 | 34100.6 | -23 | 6.50% | F1 | 28 | 23 | 1 | 11 |
| 33516.8 | 33516.6 | -6 | 6.40% | F1 | 26 | 21 | 2 | 11 |
| 33476.8 | 33476.5 | -9 | 6.00% | F2 | 27 | 22 | 1 | 10 |
| 36029.1 | 36029 | -3 | 6.00% | F2 | 30 | 25 | 1 | 15 |
| 34207.5 | 34207.6 | 3 | 5.80% | F2 | 29 | 24 | 1 | 10 |
| 33696 | 33696.5 | 15 | 5.80% | F2 | 28 | 23 | 0 | 10 |
| 34060.4 | 34060.5 | 3 | 5.80% | F2 | 27 | 22 | 3 | 11 |
| 33914.2 | 33914.4 | 6 | 5.60% | F2 | 27 | 22 | 2 | 11 |
| 32239.8 | 32240.4 | 19 | 5.60% | F2 | 28 | 23 | 0 | 5 |
| 33185.6 | 33185.5 | -3 | 5.50% | F2 | 27 | 22 | 1 | 9 |
| 33373.7 | 33374 | 9 | 5.50% | F1 | 28 | 23 | 2 | 8 |
| 34570.8 | 34570.6 | -6 | 5.50% | F2 | 28 | 23 | 2 | 12 |
| 35008.2 | 35008.5 | 9 | 5.30% | S | 32 | 27 | 1 | 9 |
| 32306.8 | 32307 | 6 | 5.30% | S | 25 | 20 | 4 | 7 |
| 32602.1 | 32602.5 | 12 | 5.00% | F2 | 25 | 20 | 4 | 8 |
| 34934.2 | 34934.5 | 9 | 4.80% | S | 31 | 26 | 1 | 10 |
| 34977.1 | 34977 | -3 | 4.80% | F1 | 30 | 25 | 0 | 12 |
| 32937.4 | 32937.5 | 3 | 4.70% | F1 | 28 | 23 | 3 | 6 |
| 34424.7 | 34424.5 | -6 | 4.50% | S | 30 | 25 | 4 | 8 |
| 34425.7 | 34426 | 9 | 4.30% | F2 | 28 | 23 | 3 | 11 |
| 36685.7 | 36685.5 | -5 | 4.20% | F2 | 31 | 26 | 1 | 16 |
| 34352.6 | 34352.5 | -3 | 4.10% | F2 | 29 | 24 | 0 | 11 |
| 33404.8 | 33404.5 | -9 | 4.00% | F2 | 28 | 23 | 0 | 9 |
| 33228.6 | 33229 | 12 | 4.00% | F1 | 28 | 23 | 3 | 7 |
| 32894.3 | 32894 | -9 | 3.50% | F2 | 27 | 22 | 1 | 8 |
| 36437.5 | 36437.5 | 0 | 3.30% | F1 | 32 | 27 | 3 | 13 |
| 32821.3 | 32821 | -9 | 2.80% | F2 | 26 | 21 | 3 | 8 |
| 32646.1 | 32646.5 | 12 | 2.70% | F1 | 28 | 23 | 3 | 5 |
| 33112.5 | 33112.6 | 3 | 2.60% | F2 | 26 | 21 | 3 | 9 |
| 33006.4 | 33006.5 | 3 | 2.50% | F1 | 25 | 20 | 3 | 10 |
| 33332.8 | 33333 | 6 | 1.90% | F2 | 27 | 22 | 4 | 8 |
| 32568 | 32567.6 | -12 | 1.70% | F1 | 25 | 20 | 0 | 10 |
| 33040.5 | 33041 | 15 | 1.50% | F2 | 27 | 22 | 2 | 8 |
| 32748.2 | 32748.5 | 9 | 1.30% | F2 | 27 | 22 | 0 | 8 |
| 34246.5 | 34247 | 15 | 1.30% | F1 | 28 | 23 | 0 | 12 |
| 32676.2 | 32676 | -6 | 1.10% | F2 | 26 | 21 | 4 | 7 |
| 32454.9 | 32454.5 | -12 | 0.80% | F2 | 25 | 20 | 1 | 9 |
| 32310.8 | 32311 | 6 | 0.70% | F2 | 25 | 20 | 4 | 7 |
| 34976.2 | 34975.6 | -17 | 0.50% | F1 | 28 | 23 | 3 | 13 |
| 34392.6 | 34392.6 | 0 | 0.30% | F1 | 28 | 23 | 1 | 12 |

^a^Relative intensity based on the intensities of the intact masses after deconvolution.

^b^total number of hexoses (Hex), N-acetylhexosamine (HexNAc), fucose (Fuc) and sialic acids (SA)

Table S11. Putatively assigned AGP glycoforms from 1:20 diluted DBS eluate by nano-CZE-MS cut 3 (ordered by decreasing relative intensity).

| Theoretical Mass [Da] | Observed Mass [Da] | Deviation [ppm] | Rel. Int^a^ | Variant | Glycosylation^b^ | | | |
| --- | --- | --- | --- | --- | --- | --- | --- | --- |
|  |  |  |  |  | Hex | HexNac | Fuc | SA |
| 35490.6 | 35490.5 | -3 | 100.00% | F1 | 33 | 28 | 0 | 10 |
| 35198.4 | 35198.5 | 3 | 84.00% | F1 | 31 | 26 | 3 | 10 |
| 35153.4 | 35153.9 | 14 | 71.80% | S | 32 | 27 | 0 | 10 |
| 33812.1 | 33812.4 | 9 | 66.20% | F1 | 30 | 25 | 0 | 8 |
| 34615.9 | 34616 | 3 | 60.60% | F1 | 31 | 26 | 3 | 8 |
| 35371.6 | 35372 | 11 | 54.00% | S | 31 | 26 | 2 | 11 |
| 34906.1 | 34906.6 | 14 | 50.20% | F1 | 31 | 26 | 1 | 10 |
| 35563.7 | 35563.5 | -6 | 45.70% | F1 | 32 | 27 | 3 | 10 |
| 35272.5 | 35272 | -14 | 44.30% | F1 | 32 | 27 | 3 | 9 |
| 34790 | 34790 | 0 | 37.60% | F2 | 29 | 24 | 1 | 12 |
| 35124.3 | 35124 | -9 | 30.00% | F1 | 30 | 25 | 3 | 11 |
| 35051.2 | 35051 | -6 | 28.80% | F1 | 31 | 26 | 0 | 11 |
| 35636.8 | 35637 | 6 | 26.90% | F1 | 33 | 28 | 1 | 10 |
| 34320.6 | 34321 | 12 | 25.90% | F1 | 29 | 24 | 0 | 11 |
| 34833.1 | 34833.5 | 11 | 25.00% | F1 | 30 | 25 | 3 | 10 |
| 34978.2 | 34978.5 | 9 | 22.60% | F1 | 30 | 25 | 2 | 11 |
| 35158.4 | 35158.5 | 3 | 22.00% | F2 | 32 | 27 | 2 | 9 |
| 35417.6 | 35418.1 | 14 | 20.60% | F1 | 32 | 27 | 2 | 10 |
| 34468.7 | 34468.5 | -6 | 19.40% | F1 | 31 | 26 | 0 | 9 |
| 34103.4 | 34103.5 | 3 | 18.60% | F1 | 30 | 25 | 0 | 9 |
| 35562.7 | 35562.5 | -6 | 18.00% | F1 | 32 | 27 | 1 | 11 |
| 33594 | 33593.5 | -15 | 16.20% | F1 | 29 | 24 | 3 | 7 |
| 34760 | 34760 | 0 | 15.60% | F1 | 31 | 26 | 0 | 10 |
| 34029.3 | 34028.9 | -12 | 14.90% | F1 | 29 | 24 | 0 | 10 |
| 35080.3 | 35080.5 | 6 | 14.70% | S | 31 | 26 | 2 | 10 |
| 35416.6 | 35416.4 | -6 | 13.10% | F1 | 32 | 27 | 0 | 11 |
| 34793.1 | 34792.6 | -14 | 12.80% | F2 | 31 | 26 | 2 | 9 |
| 34501.8 | 34501.6 | -6 | 11.90% | F2 | 31 | 26 | 2 | 8 |
| 34426.7 | 34427 | 9 | 11.10% | F2 | 30 | 25 | 0 | 10 |
| 34685.9 | 34686 | 3 | 11.10% | F1 | 30 | 25 | 0 | 11 |
| 34249.5 | 34250 | 15 | 10.80% | F1 | 30 | 25 | 1 | 9 |
| 35082.3 | 35082.6 | 9 | 10.10% | F2 | 29 | 24 | 3 | 12 |
| 34497.8 | 34497.6 | -6 | 9.90% | S | 31 | 26 | 2 | 8 |
| 33885.2 | 33885 | -6 | 9.50% | F1 | 29 | 24 | 3 | 8 |
| 34713.9 | 34713.7 | -6 | 9.20% | S | 30 | 25 | 0 | 11 |
| 34541.8 | 34542.1 | 9 | 9.20% | F1 | 30 | 25 | 3 | 9 |
| 34175.4 | 34175.5 | 3 | 8.90% | F1 | 29 | 24 | 1 | 10 |
| 33447.8 | 33447.5 | -9 | 8.70% | F1 | 29 | 24 | 2 | 7 |
| 35447.6 | 35447.8 | 6 | 8.50% | F2 | 30 | 25 | 3 | 12 |
| 33958.3 | 33958.5 | 6 | 8.30% | F1 | 30 | 25 | 1 | 8 |
| 33738 | 33738.5 | 15 | 8.30% | F1 | 29 | 24 | 0 | 9 |
| 34720 | 34720.5 | 14 | 6.90% | F2 | 30 | 25 | 4 | 9 |
| 33520.9 | 33521 | 3 | 6.60% | F1 | 30 | 25 | 0 | 7 |
| 34209.5 | 34209.1 | -12 | 6.20% | F2 | 31 | 26 | 0 | 8 |
| 34031.4 | 34032 | 18 | 5.70% | F1 | 29 | 24 | 4 | 8 |
| 33081.5 | 33081.6 | 3 | 5.50% | F1 | 28 | 23 | 0 | 8 |
| 33739.1 | 33739.5 | 12 | 5.10% | F1 | 29 | 24 | 2 | 8 |
| 34832 | 34832.5 | 14 | 5.10% | F1 | 30 | 25 | 1 | 11 |
| 34322.6 | 34322.5 | -3 | 5.00% | F1 | 29 | 24 | 4 | 9 |
| 32791.2 | 32791 | -6 | 5.00% | F1 | 28 | 23 | 2 | 6 |
| 34135.4 | 34135 | -12 | 5.00% | F2 | 30 | 25 | 0 | 9 |
| 33842.2 | 33842.5 | 9 | 4.90% | F2 | 28 | 23 | 1 | 10 |
| 34394.6 | 34394.5 | -3 | 3.80% | F1 | 30 | 25 | 0 | 10 |
| 34858.1 | 34858.5 | 11 | 2.50% | S | 28 | 23 | 2 | 13 |
| 33155.5 | 33155.9 | 12 | 2.40% | F1 | 29 | 24 | 0 | 7 |
| 33301.7 | 33302 | 9 | 1.10% | F1 | 29 | 24 | 1 | 7 |

^a^Relative intensity based on the intensities of the intact masses after deconvolution.

^b^total number of hexoses (Hex), N-acetylhexosamine (HexNAc), fucose (Fuc) and sialic acids (SA)
